# Metabolic Heterogeneity in High-Grade Glioma Assessed by Multi-Tracer PET and Ex Vivo Metabolomics: A Systematic Review and Meta-Analysis

**DOI:** 10.3390/metabo16010017

**Published:** 2025-12-24

**Authors:** Julien Todeschi, Hélène Cebula, Caroline Bund, Izzie-Jacques Namer

**Affiliations:** 1 Service de Neurochirurgie, Hôpitaux Universitaires de Strasbourg (HUS), 67200 Strasbourg, France; julien.todeschi@chru-strasbourg.fr (J.T.); helene.cebula@chru-strasbourg.fr (H.C.); 2 ICube, Université de Strasbourg/CNRS (UMR 7357), 67200 Strasbourg, France; c.bund@icans.eu; 3 Service de Médecine Nucléaire et d’Imagerie Moléculaire, Institut de Cancérologie Strasbourg Europe (ICANS), 67200 Strasbourg, France; 4 Centre Paul Strauss, 67000 Strasbourg, France

**Keywords:** high-grade glioma, positron emission tomography (PET), amino acid PET (MET/FET/FDOPA), pseudoprogression, treatment-related change, MALDI mass spectrometry imaging (MALDI-MSI), high-resolution magic angle spinning nuclear magnetic resonance (HR-MAS NMR) spectroscopy

## Abstract

**Background/Objectives**: High-grade glioma shows marked metabolic heterogeneity. We performed a PRISMA-guided systematic review and meta-analysis to quantify PET accuracy for pseudoprogression (PsP) and for recurrence/progression versus treatment-related change (TRC), assess pool baseline associations with overall (OS) and progression-free survival (PFS), summarize PET-based prediction of molecular markers, and assess the PET–stereotactic biopsy–ex vivo metabolomics workflow. **Methods**: We searched PubMed/MEDLINE and the Web of Science Core Collection (Clarivate) from inception to 1 September 2025 for HGG cohorts with baseline PET. Eligibility: Adults with HGG; diagnostic syntheses required per-patient 2 × 2; prognostic syntheses required for HR with 95% CI. Risk of bias: QUADAS-2 (diagnostic) and QUIPS (prognostic). Random-effects models pooled log-HRs and sensitivity/specificity; molecular studies were summarized by AUCs. Imaging-to-omics concordance was reviewed narratively owing to the absence of co-registered PET–metabolite maps in human HGG. **Results**: The results included the following: OS k = 10; PFS k = 3; PsP k = 2 (N = 76); and TRC k = 3 (N = 152). For PsP, two amino acid PET cohorts yielded a sensitivity of 0.943 and a specificity of 0.826. For TRC, pooled FDOPA across two cohorts gave rise to a sensitivity of 0.879 and a specificity of 0.771. OS meta-analyses were non-significant under Hartung–Knapp modification—FDG HR of 1.09 (95% CI 0.69–1.73) and amino acid HR of 1.03 (0.72–1.46)—with substantial heterogeneity. PFS effects varied by tracer/metric; examples include FDOPA HR of 7.92 (2.17–28.90) and MET metabolic tumor volume HR of 1.60 (1.20–2.30). **Conclusions**: Amino acid PET is sensitive to PsP and, with FDOPA, aids TRC discrimination when MRI is equivocal, whereas baseline PET–survival associations are weak and heterogeneous. Prospective co-registered PET/MR with stereotactic biopsies and HR-MAS NMR spectroscopy/MALDI-MSI is required to quantify imaging-to-omics concordance and standardize spatial endpoints. Study registration: PROSPERO CRD420251113416. Funding: none.

## 1. Introduction

Glioblastoma (GBM) remains the deadliest primary brain tumor in adults, with 5-year survival <7% despite maximal surgery and chemoradiotherapy [1]. Conventional MRI is essential yet imperfect: gadolinium enhancement reflects blood–brain barrier disruption rather than tumor burden, obscuring PsP and metabolic heterogeneity [2].

Positron emission tomography (PET) offers complementary metabolic insights. ^18^F-FDG, though limited by cortical background, predicts outcome based on tumor-to-normal-brain ratios [3,4]. Amino acid tracers—^18^F-FET, ^18^F-FDOPA, ^11^C-MET—target LAT1 transporters and improve biopsy guidance, grading, and early response monitoring [5,6,7,8]. Hypoxia agents (^18^F-FMISO and ^18^F-FAZA) delineate radio-resistant areas; in GBM, a higher ^18^F-FMISO hypoxic burden has been associated with worse outcomes (FAZA in HGG [9,10]; FMISO outcome [11]). Emerging receptor-targeted tracers such as ^68^Ga-Pentixafor (CXCR4) or ^68^Ga-PSMA highlight aggressive or angiogenic subregions [12,13,14].

Ex vivo HR-MAS ^1^H NMR spectroscopy and MALDI-MSI map micro-regional metabolic gradients in GBM (elevated choline/lactate, lipid heterogeneity) [15,16]. In vivo ^1^H-MRS detects 2-hydroxyglutarate in IDH-mutant gliomas [17]. These metabolomic maps inform histopathology and, when combined with in vivo imaging (e.g., amino acid PET on hybrid PET/MR), can refine target-volume delineation and help expose drivers of treatment escape [18,19].

Beyond diagnostic and prognostic aims, this review explicitly foregrounds the triangulation of multimodal PET (amino acid, hypoxia, receptor-targeted), stereotactic biopsy guidance, and ex vivo metabolomics (HR-MAS NMR spectroscopy/MALDI-MSI) to link in vivo metabolic “hotspots” to tissue-resolved signatures—an approach aligned with recent RANO-EANO recommendations and EANM/EANO/SNMMI guidelines [20,21].

Recent guidelines underscore this need. The joint EANM/EANO/RANO/SNMMI guidelines advocate for amino acid PET whenever MRI is inconclusive, emphasizing its superiority for treatment monitoring [21]. Likewise, the 2025 RANO–EANO policy review highlighted that multi-tracer PET combined with radiomics could capture the metabolic mosaic of GBM, yet it is limited by the paucity of systematic evidence [20]; see MET-PET/MRI radiomics in GBM [22]. Taken together, these statements point to a critical knowledge gap: how do glycolytic, amino acid, hypoxia, and receptor-targeted tracers compare and to what extent do ex vivo spectrometric studies validate their in vivo signatures?

To our knowledge, no previous review has combined diagnostic accuracy, prognostic meta-analysis, and imaging-to-omics concordance across multi-tracer PET and ex vivo spectrometry in high-grade glioma. We aim to pool survival HRs across FDG, amino acid, hypoxia, and receptor-targeted PET; summarize diagnostic accuracy for IDH status and PsP; assess PET–metabolite concordance; and identify gaps for future hybrid imaging. Study registration: PROSPERO CRD420251113416.

## 2. Materials and Methods

### 2.1. Study Design and Registration

This study was a PRISMA-2020 systematic review and meta-analysis (PROSPERO CRD420251113416). We prespecified four objectives. First (prognostic), we evaluated baseline associations between PET metrics and overall survival (OS) or progression-free survival (PFS) in adults with high-grade glioma (HGG, WHO III–IV). Second (diagnostic), we assessed the accuracy of PET for pseudoprogression (PsP) after chemoradiotherapy and for recurrence/progression versus treatment-related change (TRC). Third (molecular), we summarized PET-based prediction of molecular markers (IDH, MGMT, 1p/19q, ATRX, TERT). Fourth (imaging-to-omics), we reviewed concordance between in vivo PET and ex vivo metabolomics (HR-MAS NMR spectroscopy, MALDI-MSI). Only the prognostic and diagnostic components were eligible for quantitative meta-analysis; molecular and imaging-to-omics components were synthesized qualitatively because of heterogeneous endpoints and reporting.

### 2.2. Eligibility

Adults with high-grade glioma (HGG, WHO grade III–IV) were eligible. We excluded studies with a total sample size <10, purely pediatric cohorts, purely low-grade glioma cohorts without a separable HGG subgroup, preclinical animal or phantom work, qualitative SPECT-only imaging, narrative reviews, case reports, and studies missing key statistics required for any of the planned syntheses.

For diagnostic accuracy, we required per-patient 2 × 2 data (true positives, false positives, false negatives, true negatives) for pseudoprogression (PsP) after chemoradiotherapy and/or for recurrence/progression versus treatment-related change (TRC). The reference standard was histopathology when reoperation or biopsy was performed, or standard-of-care clinical/radiological follow-up according to RANO or Macdonald criteria when histology was not available. Mixed reference standards within a cohort (histology for some patients, RANO-based follow-up for others) were allowed when they reflected routine clinical practice and were clearly described. Studies that only reported per-lesion analyses without per-patient data were excluded from quantitative diagnostic pooling.

For prognostic analyses, eligible designs were prospective or retrospective cohort studies and randomized trials in which baseline PET metrics were analyzed as prognostic factors for overall survival (OS) and/or progression-free survival (PFS). We recorded the line of therapy (newly diagnosed versus recurrent), prior treatments when reported (e.g., bevacizumab), and median follow-up. Studies were excluded from quantitative pooling if they did not report a hazard ratio (HR) with a 95% confidence interval (CI) or if PET was used solely to define treatment groups without a separate baseline prognostic analysis. When substantial baseline imbalances between PET-defined groups were present, we only retained HRs from multivariable models that adjusted at least for age and performance status and MGMT promoter methylation and/or extent of resection when available.

For prognostic pooling, PET-guided interventional and extent-of-resection (EOR) studies were excluded when the PET metric of interest directly determined treatment allocation or intensification and no separate baseline prognostic analysis was available (“inseparable” designs). Interventional studies were retained when baseline PET was analyzed as an independent prognostic factor (e.g., as a covariate in multivariable models that did not dictate treatment allocation).

### 2.3. Information Sources and Search

We searched PubMed/MEDLINE (NCBI) and the Web of Science Core Collection (Clarivate) from inception to 1 September 2025. No language limits were applied at the search stage; non-English records were screened when an English abstract was available. The search combined terms for high-grade glioma, PET tracers (FDG, amino acid, hypoxia and receptor-targeted agents), and ex vivo spectrometry (HR-MAS, MALDI-MSI). The full reproducible search strings, including MeSH terms, free-text synonyms, Boolean operators, field tags, and date limits, are provided in Supplementary Table S1. Institutional access to Embase and Scopus was not available to the authors; we therefore maximized coverage by using two complementary databases (PubMed and Web of Science), backward–forward citation chasing from key guidelines and narrative reviews, and screening of trial registries (ClinicalTrials.gov and WHO ICTRP).

**Figure 1:**
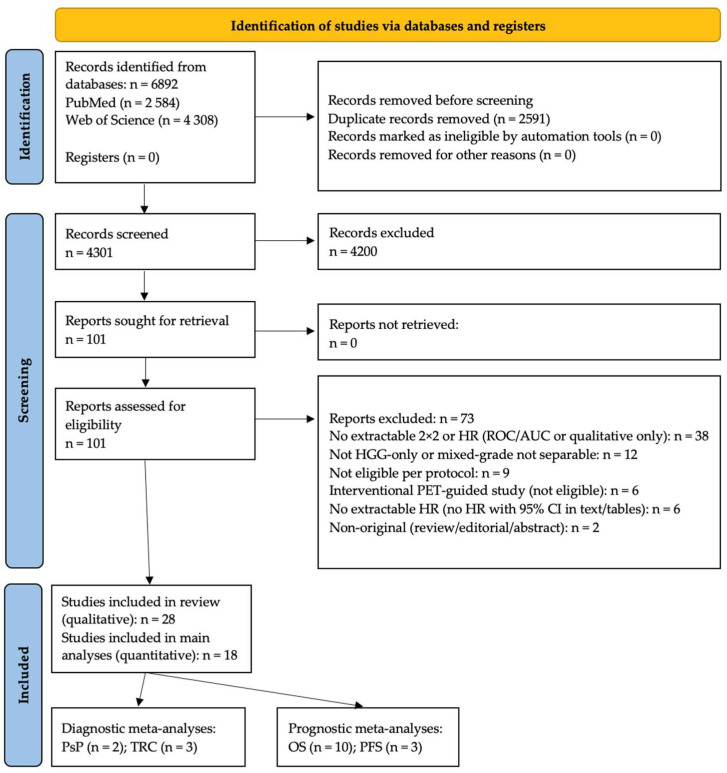
PRISMA 2020 flow diagram of study selection; databases searched: PubMed and Web of Science (Clarivate); key counts as reported in Methods.

The PubMed query was (GBM OR “HGG”) AND (FDG OR FET OR FDOPA OR MET OR FMISO OR FAZA OR Pentixafor OR PSMA OR “HR MAS” OR MALDI); an equivalent Topic (TS) search was run in Web of Science. No language limits were imposed on the search (screening required an English abstract). We performed backward–forward citation chasing, screened ClinicalTrials.gov and WHO ICTRP, exported full records, and deduplicated studies, first by DOI and then by normalized title, prior to screening. Study selection followed PRISMA 2020; the flow diagram with all counts is shown in Figure 1.

### 2.4. Data Extraction

Two reviewers independently screened full texts and extracted data into piloted structured spreadsheets (one template for diagnostic/prognostic PET data and one for ex vivo metabolomics), with discrepancies resolved by consensus; no automation tools were used. For all eligible studies, we recorded design (prospective/retrospective cohort or trial), sample size, HGG grade distribution, key molecular markers (IDH, MGMT, 1p/19q, ATRX, TERT) when reported, clinical context (newly diagnosed versus recurrent disease), treatment era, prior systemic therapies (including bevacizumab), and the timing of PET relative to surgery and chemoradiotherapy (baseline, post-CRT, or surveillance). For each PET dataset, we abstracted tracer, acquisition protocol, timing, reconstruction, and the quantitative metrics reported (SUV, TBR, MTV, TLG, dynamic parameters, radiomics scores).

For diagnostic accuracy studies, we extracted per-patient 2 × 2 tables (true positives, false positives, false negatives, true negatives) for each relevant tracer–endpoint combination (PsP after chemoradiotherapy; recurrence/progression versus TRC), as well as the positivity threshold (e.g., TBRmax cut-off) and the type of reference standard used (histology versus RANO/Macdonald-based follow-up). When multiple thresholds were reported, we retained the threshold used in the main analysis or the one with the most clinically relevant balance of sensitivity and specificity.

For prognostic studies, we extracted HRs with 95% CIs for OS and/or PFS, together with the type of model (univariable versus multivariable) and all covariates included in adjusted models (e.g., age, performance status, MGMT, IDH, extent of resection, treatment variables). When both unadjusted and adjusted HRs were reported for the same PET metric and endpoint, we retained the adjusted estimate. When several PET metrics were analyzed within the same study, we retained a single HR per study–endpoint according to a prespecified tracer-specific hierarchy (favoring volumetric or dynamic metrics over purely static uptake measures when no primary metric was specified). All extracted HRs, confidence intervals, model types, and covariates are listed in Supplementary Table S6.

### 2.5. Risk of Bias

We used QUADAS-2 (diagnostic, including applicability concerns) and QUIPS (prognostic) reported in Supplementary Table S3 (QUADAS-2) and Supplementary Table S4 (QUIPS). Two reviewers independently applied QUADAS-2 to diagnostic studies (low/high/unclear) and QUIPS to prognostic cohorts (low/moderate/high; unclear when reporting was insufficient); disagreements were resolved by consensus. Study-level judgments are reported in Supplementary Table S3 (QUADAS-2) and Supplementary Table S4 (QUIPS). Sensitivity analyses (e.g., excluding high-risk studies) were informed by these judgments (QUADAS-2 for diagnostic accuracy; QUIPS with low/moderate/high categories for prognosis). We did not apply a formal GRADE rating of certainty because the evidence base comprised small, heterogeneous, and largely retrospective cohorts; instead, we relied on QUADAS-2 and QUIPS assessments combined with sensitivity analyses and Hartung–Knapp-adjusted random-effects models to convey uncertainty.

### 2.6. Effect Measures and Synthesis

Diagnostic accuracy was summarized using study-level 2 × 2 tables (true positives, false positives, false negatives, true negatives). For pseudoprogression (PsP) after chemoradiotherapy and for recurrence/progression versus treatment-related change (TRC), we prespecified bivariate hierarchical summary receiver-operating characteristic (HSROC) models when at least three comparable per-patient datasets were available within a given tracer/outcome stratum. In practice, only two amino acid PET cohorts contributed per-patient PsP data and only two ^18^F-FDOPA cohorts contributed TRC data, with one additional small cohort for FDG and 13N-NH3. Given this small k and tracer heterogeneity, we did not fit HSROC models. Instead, within each stratum we aggregated raw 2 × 2 totals to obtain simple pooled sensitivity and specificity with binomial 95% confidence intervals; these summary points are therefore descriptive rather than formal HSROC estimates. For PsP we categorized ^18^F-FDOPA and ^18^F-fluciclovine as amino acid PET, and given the small k and partially non-identical tracers, the pooled estimates should be interpreted with caution. When any study-level 2 × 2 cell was equal to zero, we applied a 0.5 continuity correction in sensitivity analyses for variance estimation only; raw 2 × 2 counts and aggregated totals are reported without correction. All per-patient 2 × 2 tables and zero-cell flags are provided in Supplementary Table S5. For prognostic outcomes (overall survival [OS] and progression-free survival [PFS]), the effect measure was the hazard ratio (HR). HRs and 95% confidence intervals were transformed to log(HR) and corresponding standard errors, and random-effects meta-analyses were performed using restricted maximum likelihood (REML) with Hartung–Knapp adjustment. We prespecified that covariate meta-regression would only be attempted when at least ten studies contributed to a given prognostic stratum; this threshold was not met (k = 4 for FDG and k = 4 for amino acid PET), so no formal meta-regression was performed. Instead, we used leave-one-out and influence analyses to assess robustness and report Hartung–Knapp-adjusted prediction intervals. Because no prognostic stratum included ten or more studies, we did not perform formal tests for small-study effects (e.g., funnel plots or regression-based asymmetry tests). Given the heterogeneity of PFS endpoints and PET metrics, we did not pool PFS across tracers but report study-level estimates by tracer and metric. Molecular marker prediction and PET–metabolite concordance outcomes were synthesized qualitatively because of heterogeneous endpoints, small k, and the absence of voxel-wise co-registered PET–metabolite maps in human HGG.

## 3. Results

### 3.1. PsP vs. True Progression—Per-Patient (HGG)

Across two per-patient amino acid PET cohorts—one with ^18^F-FDOPA [23] and one with ^18^F-FACBC [24] —diagnostic performance clustered in a high and consistent range. A simple aggregation over 2 × 2 totals (AA-PET-pooled) yielded a sensitivity of 0.943 (50/53) and a specificity of 0.826 (19/23) (N = 76). Individually, FDOPA and FACBC showed near-identical operating points (0.935/0.824 [23]; 0.955/0.833 [24]), suggesting that—despite differences in protocols and a small k—amino acid tracers offer similar discriminative ability for PsP in HGG. Given only two cohorts and heterogeneous tracers, we refrain from model-based meta-analysis and report descriptive study-level values alongside the pooled summary (Table 1).

For PsP (two amino acid PET cohorts; N = 76), the aggregated 2 × 2 data yielded a sensitivity of 0.94 (50/53) and a specificity of 0.83 (19/23). Given k = 2 and tracer heterogeneity (FDOPA versus fluciclovine), these estimates should be interpreted as descriptive rather than as formal HSROC outputs.

### 3.2. Recurrence/Progression vs. Treatment-Related Change (TRC)—Per-Patient, HGG-Only (Main Analysis)

Restricting the analysis to per-patient HGG cohorts with explicit 2 × 2 data identified three eligible studies (four tracer arms). Within the FDOPA subgroup (Herrmann 2014 [25]; Karunanithi 2013 [26]), pooled patient-level counts gave a sensitivity of 0.879 (87/99) and a specificity of 0.771 (27/35) (N = 134). In a study by Khangembam, 2014 [27], ^18^F-FDG and ^13^N–NH_3_ were evaluated on the same 18-patient HGG cohort, each arm yielding a sensitivity of 0.778 and a specificity of 0.667 (per arm N = 18; unique N unchanged). Given the small number of studies and tracer heterogeneity, we do not fit a single HSROC and instead present study-level results, complemented by the FDOPA subgroup summary (Table 2). Per-lesion FET series are referenced narratively to avoid unit-of-analysis bias.

For TRC with FDOPA (two cohorts; N = 134), aggregated counts gave a sensitivity of 0.88 (87/99) and a specificity of 0.77 (27/35); again, with k = 2, these estimates are descriptive.

QUADAS-2 judgments are provided in Supplementary Table S3; the main concerns were patient selection and threshold specification, with generally acceptable reference standards and flow/timing.

### 3.3. Overall Survival (OS) and Progression-Free Survival (PFS)

We synthesized 10 overall survival (OS) estimates (FDG: k = 4; amino acid tracers [FET/FDOPA/MET]: k = 4; emergent tracers FLT and FMISO: one study each) and 3 progression-free survival (PFS) estimates (see Table 3). Random-effects pooling for OS showed modest or null associations with substantial heterogeneity.

**Figure 2:**
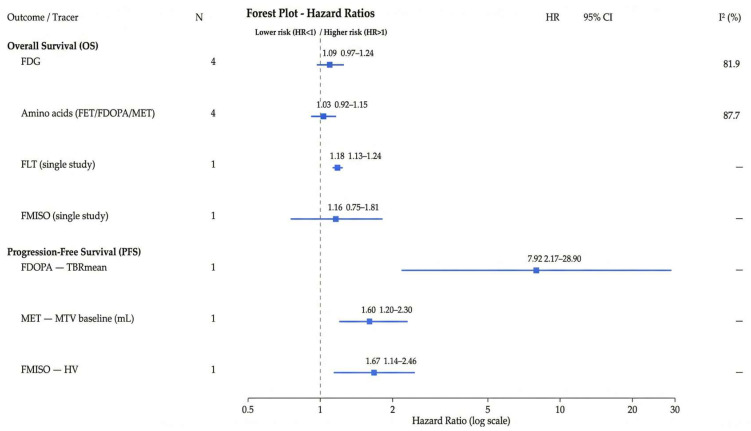
Forest plot of hazard ratios (OS and PFS). Legend—squares indicate point estimates; horizontal bars show 95% CIs; the dashed vertical line marks HR = 1. Blue denotes OS: large squares = pooled estimates (FDG; amino acid PET), small squares = single-study estimates (FLT, FMISO). Red denotes PFS single-study estimates (FDOPA, MET, FMISO). The pooled amino acid OS includes Jansen 2015 [28] (dynamic TTP), per protocol. *X*-axis on log scale. Abbreviations: TBR, tumor-to-background ratio; MTV, metabolic tumor volume; HV, hypoxic volume. Numerical values and covariate sets for all hazard ratios shown in this figure are provided in Supplementary Table S6.

For FDG (k = 4), the pooled HR was 1.09 (95% CI 0.97–1.24; I^2^ = 81.9%). For amino acid tracers (k = 4), the pooled HR was 1.03 (95% CI 0.92–1.15; I^2^ = 87.7%). As prespecified, the amino acid pooled OS analysis included Jansen 2015 [28], who used a dynamic metric (time-to-peak, TTP); sensitivity analyses excluding dynamic metrics did not materially change the pooled estimate (see Figure 2).

Using REML with Hartung–Knapp modification as the primary estimator yielded wider uncertainty: FDG HR of 1.09 (95% CI 0.69–1.73; 95% PI 0.53–2.26) and amino acid HR of 1.03 (95% CI 0.72–1.46; 95% PI 0.57–1.85).

Emergent tracers were not pooled; we report single-study effects: FLT HR of 1.18 (95% CI 1.13–1.24) and FMISO HR of 1.16 (95% CI 0.75–1.81).

For PFS, we did not pool across amino acid tracers due to metric/tracer heterogeneity; study-level effects were FDOPA (Rozenblum 2023 [29]; TBRmean) HR of 7.92 (95% CI 2.17–28.90) and MET (Miller 2020 [30]; MTV per 10 mL) HR of 1.60 (95% CI 1.20–2.30). For emergent tracers, FMISO (Huang 2021 [31]; hypoxic volume) HR was 1.67 (95% CI 1.14–2.45).

Taken together, the pooled OS associations for FDG and amino acid tracers were small and statistically non-significant under HK-adjusted random effects, with wide prediction intervals spanning no effect; study-level PFS associations varied by tracer and metric.

**Table 3:** Summary of primary meta-analytic results. * Primary model: REML with Hartung–Knapp modification. The DL results shown are sensitivity analyses and align with the forest plots and the summary table. Biological coherence was maintained by separating FLT and FMISO and avoiding cross-tracer pooling for PFS.

Outcome	Tracer/Class	Studies (Years)	k	Model	HR	95% CI	I^2^ (%)
**OS**	FDG	Colavolpe (2012) [32]; Leiva-Salinas (2017) [33]; Chiang (2017) [34]; Graham (2020) [35]	4	Random-effects (HK-REML) *	1.09	0.97–1.24	81.9
**OS**	Amino acid (FET/FDOPA/MET)	Jansen (2015) [28]; Suchorska (2015) [36]; Bauer (2020) [37]; Wirsching (2021) [38]	4	Random-effects (HK-REML) *	1.03	0.92–1.15	87.7
**OS**	FLT	Zhao (2014) [39]	1	—	1.18	1.13–1.24	—
**OS**	FMISO	Gerstner (2016) [11]	1	—	1.16	0.75–1.81	—
**PFS**	FDOPA—TBRmean	Rozenblum (2023) [29]	1	—	7.92	2.17–28.90	—
**PFS**	MET—MTV (per 10 mL)	Miller 2020 [30]	1	—	1.60	1.20–2.30	—
**PFS**	FMISO—hypoxic volume	Huang 2021 [31]	1	—	1.67	1.14–2.45	—

Overall QUIPS was low-to-moderate; down-weighting/excluding high-risk studies did not materially change pooled estimates. Details are provided in Supplementary Table S4.

### 3.4. Molecular Markers (Study-Level Synthesis)

We summarized diagnostic performance for IDH, MGMT, ATRX, and TERT from amino acid PET and PET radiomics. Owing to heterogeneous endpoints/reporting (AUC vs. Se/Sp; static vs. dynamic; mono- vs. multimodal), no pooling was performed. Per-study data are provided in Table 4; distributional summaries are given in Table 5.

Across eight IDH studies, performance was consistently high (median AUC 0.881, IQR 0.807–0.917). Strong results appeared with both handcrafted features and radiomics across FET, FDOPA, and MET, including multimodal PET + MRI (e.g., DSC-PWI). Single-center PET/MR and radiomics pipelines reached AUCs of up to 0.97, while conventional static metrics remained robust (≈0.86–0.90).

MGMT showed moderate discrimination overall (median AUC ≈ 0.80 across two studies): one PET/MR study yielded perfect specificity but lower sensitivity; a static FET study reported AUC 0.706. Methodological variability (PET/MR vs. PET/CT; volumetric thresholds vs. learned models) likely drives dispersion; larger, harmonized datasets are needed.

For ATRX, a single static FET study reported an AUC of 0.736. TERT ranged from 0.674 (MET) to 0.860 (FET PET/MR), indicating moderate, tracer-/pipeline-dependent performance.

Given scarce per-patient 2 × 2 data and heterogeneity, pooling risked bias, we therefore report medians/IQRs in the text and defer cutoffs/models to Table 4. Practically, IDH prediction is already strong across tracers and modeling strategies. MGMT is promising but variable. ATRX and TERT evidence is sparse, with moderate AUCs suggesting room for improvement via optimized features and multimodal PET/MR.

**Table 4:** Per-study extraction for molecular marker prediction. Per-study extractions listing tracer, endpoint, sample size, and performance metrics (Se, Sp, accuracy, AUC), along with the applied cutoff or model (e.g., TBR_max, volumetric CU ratio, PET/MR radiomics, multimodal fusion).

Marker	First Author (Year)	Tracer	Endpoint	N_Total	Se	Sp	Acc	AUC	Cutoff/Model
**IDH**	Bai (2025) [40]	^18^F-FET (PET/MR)	IDH mutation (prediction)	29	1.000	0.824	0.900	0.970	—
**IDH**	Kaiser (2024) [41]	^18^F-FET + ^18^F-GE-180 + MRI	IDH prediction (radiomics)	87	—	—	—	0.960	Multimodal model
**IDH**	Lohmeier (2023) [42]	^18^F-FET (static)	IDH mutation (prediction)	26	0.910	0.870	0.880	0.896	Volumetric CU ratio > 5.43
**IDH**	Zaragori (2022) [43]	^18^F-FDOPA (radiomics)	IDH mutation (prediction)	72	—	—	—	0.831	—
**IDH**	Zaragori (2021) [44]	^18^F-FDOPA (dynamic)	IDH mutation (prediction)	37	—	—	—	0.733	—
**IDH**	Zhou (2021) [45]	^11^C-MET (PET/CT radiomics)	IDH mutation (prediction)	72	—	—	—	0.866	—
**IDH**	Nakajo (2021) [46]	^11^C-MET	IDH mutation (prediction)	68	0.692	0.762	—	0.725	Mean L/N = 2.05
**IDH**	Song (2021) [47]	^18^F-FET + DSC-PWI	IDH mutation (prediction)	52	0.920	0.857	—	0.903	TBRmax 3.806 + nCBVmean 1.035
**MGMT**	Bai (2025) [40]	^18^F-FET (PET/MR)	MGMT promoter methylation	29	0.684	1.000	0.793	0.900	—
**MGMT**	Lohmeier (2023) [42]	^18^F-FET (static)	MGMT methylation	45	—	—	0.640	0.706	Volumetric CU ratio
**ATRX**	Lohmeier (2023) [42]	^18^F-FET (static)	ATRX loss	46	—	—	0.740	0.736	Volumetric CU ratio
**TERT**	Bai (2025) [40]	^18^F-FET (PET/MR)	TERT promoter mutation	29	0.714	0.875	0.759	0.860	—
**TERT**	Nakajo (2021) [46]	^11^C-MET	TERT promoter mutation	68	0.500	0.893	—	0.674	Mean L/N = 1.88

**Table 5:** Summary of study-level AUCs by molecular marker. Distributional summary of study-level AUCs per marker (median, IQR, range). Estimates are not pooled owing to heterogeneous endpoints and reporting.

Marker	k (AUC Available)	Median AUC	IQR	Min–Max
**IDH**	8	0.881	0.807–0.917	0.725–0.970
**MGMT**	2	0.803	0.706–0.900	0.706–0.900
**ATRX**	1	0.736	0.736–0.736	0.736–0.736
**TERT**	2	0.767	0.674–0.860	0.674–0.860

### 3.5. Ex Vivo Spectrometry (HR-MAS NMR Spectroscopy, MALDI-MSI) and PET Map Concordance

We screened human ex vivo studies using HR-MAS NMR spectroscopy and MALDI-MSI in cohorts including HGG. Although PET was often part of the clinical work-up, no study performed direct cartographic co-registration between PET uptake maps (FDG, FET, FDOPA, etc.) and ex vivo metabolic maps. Consequently, voxel-wise metrics—Dice/Jaccard overlap or within-lesion correlations between PET intensity and metabolite abundance—were unavailable for pooling. We therefore provide a structured per-study inventory and a narrative synthesis (Table 6).

Regarding the concordance of multimodal PET with biopsy/metabolomics, at the regional scale, ex vivo HR-MAS NMR spectroscopy consistently shows elevated choline and lipids in aggressive GBM subregions, while MALDI-MSI enables on-tissue quantification of 2-hydroxyglutarate for IDH assessment [15,48]. Biopsy-controlled hybrid PET/MR studies further confirm that amino acid PET delineates tumor hotspots beyond contrast-enhanced MRI [18,19]. However, no human HGG cohort has yet reported voxel-wise PET metabolite co-registered maps, precluding spatial meta-analysis; this gap motivates prospective PET/MR protocols with stereotactic sampling and harmonized overlap/correlation metrics.

Across GBM/HGG specimens, HR-MAS NMR spectroscopy consistently showed reproducible intra-tumoral micro-heterogeneity: lactate and mobile lipids increased near necrotic/ischemic regions, whereas choline compounds (phosphocholine, glycerophosphocholine) tracked cellularity and membrane turnover. HR-MAS NMR spectroscopy preserves tissue architecture, enabling paired histopathology. MALDI-MSI yielded complementary, high-resolution metabolite and lipid maps; multiple studies demonstrated on-tissue 2-hydroxyglutarate, supporting IDH assessment. MSI-MRI co-registration pipelines (rigid/deformable) exist, implying that MSI-PET is feasible; however, PET ex vivo registration with map-level overlap/correlation has not yet been reported in human HGG.

Overall, ex vivo platforms corroborate the biological specificity of PET “hot-spots” and “cold-spots” (e.g., choline with proliferation; lactate/lipids with hypoxia/necrosis), but the absence of co-registered cartography precludes formal concordance estimates. This gap explains our choice to forgo a spatial meta-analysis and instead summarize study-level findings (Table 6). Prospective protocols linking pre-resection PET to neuronavigation-guided sampling and post-processing MSI-MRI-PET registration would enable voxel-wise overlap and correlation analyses.

**Table 6:** Inventory of ex vivo studies (HR-MAS, MALDI-MSI) including HGG. Legend—ex vivo platform, cohort (including HGG), presence of PET imaging in the cohort, PET ex vivo co-registration, spatial metrics (Dice/Jaccard/correlation), and main findings.

First Author (Year)	Platform	Cohort (HGG Included)	Tissue Sampling (Multi-Region?)	In Vivo PET in Cohort	PET ↔ Ex Vivo Co-Registration (Map)	Spatial Metric (Dice/Jaccard/Corr)	Main Finding(s)
**Cheng (2000)** [15]	HR-MAS	GBM (Yes)	Yes (multi-region)	Not reported	No	—	Lactate and mobile lipids with necrosis; PCho/Cho with cellularity; intra-tumoral micro-heterogeneity
**Chen (2011)** [49]	HR-MAS	Neuroepithelial (including. grade III–IV: 8 AA + 3 GBM)	Yes	Not reported	No	—	HR-MAS NMR spectroscopy + pattern recognition → accuracy 87% for HGG vs. LGG
**Longuespée (2018)** [50]	MALDI-TOF	Gliomas IDH mut/wt	Yes	Not reported	No	—	Rapid detection of 2-HG on tissue (≤5 min), correlated with biochemical assay
**Lan (2021)** [48]	MALDI-MSI (quant.)	Gliomas (n = 34)	Yes	Not reported	No	—	Absolute quantification of 2-HG (cutoff ≈ 0.81 pmol/µg, Se/Sp 100% for IDH)
**Kampa (2020)** [16]	MALDI-MSI	GBM	Yes	Not reported	No	—	Spatial maps of lipides/metabolites; Registration workflows: MSI ↔ MRI (transferable to PET)

### 3.6. Risk of Bias

QUADAS-2 (diagnostic) and QUIPS (prognostic) results are shown in Supplementary Tables S3 and S4 (traffic-light heatmaps). The most frequent concerns were patient selection (non-consecutive/case–control) and post hoc index-test thresholds; reference standard and flow/timing were generally acceptable. In prognostic studies, control of confounding varied. These judgments informed the sensitivity analyses.

## 4. Discussion

### 4.1. Key Findings—Concise Summary

Baseline PET uptake showed limited standalone prognostic value for OS in HGG (pooled HRs ≈ 1.0 for FDG and amino acid tracers). In contrast, amino acid PET—especially FDOPA—yielded consistently high diagnostic accuracy for treatment-related change: pooled Se/Sp ≈ 0.94/0.83 for PsP and ≈0.86/0.75 for recurrence vs. treatment effect. Thus, amino acid PET is the preferred adjunct in equivocal post-CRT assessments, whereas baseline uptake metrics appear context-dependent for prognosis.

### 4.2. Principal Findings and Integration with Prior Work

To avoid incoherent pooling, prognostic meta-analysis was restricted to baseline, non-PET-guided associations reporting HRs with 95% CIs, stratified by tracer class. Dynamic/post-treatment metrics were excluded (with one prespecified exception for FET-TTP when no static alternative existed), and biologically distinct tracers (e.g., FLT vs. FMISO) were analyzed separately. For PFS with amino acid tracers, heterogeneous metrics (e.g., FDOPA-TBR vs. MET-MTV) were presented side-by-side rather than forced into a single pool. These choices may increase apparent I^2^ in broad aggregates but keep conclusions transparent and defensible.

Across multivariable cohorts with amino acid PET, volumetric (e.g., [^18^F]FET biological tumor volume (BTV)) and dynamic parameters (e.g., time-to-peak, slope) retained independent prognostic value after adjustment for clinical and molecular covariates (age, performance status, MGMT; surgical extent when available) [28,36,37,51,52].

Beyond glycolysis, amino acid tracers (FET, FDOPA) and hypoxia agents (FMISO/FAZA) add complementary biology: across tracers, higher SUVmax predicted shorter PFS (pooled HR 1.45, 95% CI 1.11–1.90) [53]; in recurrent GBM, greater FMISO hypoxic volume also predicted shorter PFS (HR ≈1.67) [31]. Genotype modulates FET: in newly diagnosed IDH-wild-type astrocytic gliomas, dynamic FET parameters independently predicted OS, whereas static uptake was less robust [37]. FDOPA-PET radiomics can non-invasively predict molecular markers (including IDH) with strong single-center performance, while current pooled meta-analyses remain largely MRI-based rather than FDOPA-specific [43].

Ex vivo spectrometry corroborates in vivo PET “hotspots”: choline-rich ^1^H-MRS(I) maps spatially correspond to ^18^F-FET hotspots [54]. HR-MAS NMR spectroscopy and MALDI-MSI further reveal metabolic subregions; HR-MAS NMR spectroscopy profiles (e.g., higher free choline/phosphorylcholine and lipids) are associated with poorer survival, whereas higher glutamine or creatine may be favorable [55]. Collectively, these data support a biologically grounded multi-tracer paradigm—FDG approximates global glycolysis, while amino acid and hypoxia tracers delineate invasive or radio-resistant areas; hybrid PET/MRSI can aid in precision biopsy [18,19]. Volume-based indices (MTV, TLG) and 3D habitat mapping capture whole-tumor burden and spatial heterogeneity beyond single-voxel SUVmax readouts [22]. Mechanistically, hypoxia-programmed areas upregulate HK2 and GLUT3, linking glycolytic load to aggressiveness [56,57,58]; GLUT3 suppression radiosensitizes GBM models, and genome-wide CRISPR screens identify metabolic drivers of radioresistance, underscoring the therapeutic relevance of glycolytic imaging [58,59,60].

### 4.3. Molecular Stratification and Metabolic Phenotypes

The prognostic impact of ^18^F-FDG is genotype-dependent: in IDH-wild-type GBM, greater FDG tumor burden (e.g., MTV/TLG or tumor-to-white-matter ratio) is associated with markedly shorter OS, whereas SUVmax alone is less robust [61]. Conversely, IDH-mutant gliomas show attenuated FDG avidity via 2-hydroxyglutarate-driven PDK3 up-regulation and pyruvate–dehydrogenase inhibition [62]. EGFRvIII-positive tumors can fuel mTORC2 signaling from glucose or acetate; concordantly, gliomas oxidize acetate in vivo and are visualizable with [^11^C]acetate PET [63,64]. These data support genotype-aware thresholds and favor volumetric FDG metrics in integrative prognostic nomograms.

### 4.4. Amino Acid PET and Radiomics

Amino acid PET accurately distinguishes true progression from treatment-related change; meta-analyses report pooled sensitivities of ~0.84 for FET and up to 0.95 for FDOPA with specificities of ~0.75–0.90, complementing advanced MRI [65,66,67]. For prognosis, higher SUV_max is associated with shorter PFS (pooled HR ~1.45) [53]. Radiomics further refines molecular stratification: in a 274-patient multi-center study, FDOPA-PET radiomics identified IDH-mutant gliomas (AUC 0.83), and a prospective 57-patient dynamic FDOPA study with point-spread-function deconvolution achieved an AUC of 0.83 for IDH/1p19q [43,65]. Consistently, reviews of reirradiation in relapsing GBM highlight the contribution of PET/MRI and radiomics to the evaluation of the peritumoral zone and to the distinction between progression and treatment-related changes [68]. Standardization remains crucial for reproducibility (IBSI; EARL harmonization) [69].

### 4.5. Hypoxia and Receptor-Targeted Tracers Delineate Therapeutic Niches

Hypoxia tracers (^18^F-FMISO, ^18^F-FAZA) identify radio-/chemo-resistant subvolumes; in recurrent GBM, a larger FMISO-defined hypoxic volume predicts earlier progression (HR ≈ 1.67) [31]. Hypoxia-guided dose-painting is under prospective evaluation (e.g., FMISO-informed RT trials) [NCT00902577; NCI-2021-08286]. Receptor tracers broaden phenotyping: ^68^Ga-Pentixafor (CXCR4) visualizes a CXCR4-high subset and supports theranostics ([^177^Lu]-Pentixather) [12,70]. ^68^Ga-PSMA shows endothelial/vascular expression and is being studied for PSMA-directed radioligand therapy. Emerging probes of glutamine uptake (^18^F-FGln) and redox balance (^18^F-FSPG) have entered human imaging, including glioma cohorts [71,72].

### 4.6. Multimodal Validation with HR-MAS NMR Spectroscopy and MALDI-MSI

Ex vivo HR-MAS NMR spectroscopy demonstrates regional metabolic heterogeneity and links metabolite levels to histopathology, enhancing the biological interpretability of in vivo PET [15]. MALDI-MSI enables on-tissue 2-HG quantification for IDH assessment, bridging imaging and molecular pathology [48]. Although direct voxel-wise PET ex vivo co-registration in human HGG is scarce, existing MSI-MRI frameworks in glioma outline a path toward prospective PET/MR with stereotactic sampling. The single most important gap is the absence of human HGG datasets with voxel-wise co-registered PET and ex vivo metabolite maps, which currently precludes any spatial meta-analysis of PET–metabolite concordance and limits quantitative validation of metabolic “hot spots”.

### 4.7. What the Ex Vivo Evidence Means for Hybrid PET/MR

Ex vivo spectrometry confirms biological heterogeneity in GBM/HGG (HR-MAS) and provides high-resolution metabolic maps (MALDI-MSI), but map-level PET concordance in human cohorts has not yet been shown. Voxel-wise PET–metabolite concordance in humans remains challenging for three practical reasons: small targeting/sampling errors around the stereotactic trajectory (and potential brain shift), tissue deformation between fresh tissue, cryosections and MSI, and cross-modality registration across PET, MRI and histology/MSI. A pragmatic workflow is to log the multimodal stereotactic trajectory and align the specimen (ink + photographs), perform rigid → deformable registration back to pre-op MRI, and sample PET within a 2–3 mm spherical kernel around the biopsy centroid. HR-MAS is compared at the centroid, whereas MALDI-MSI provides pixel-level maps, with agreement reported via spatial overlap (Dice/Jaccard) and local voxel-wise correlations (Fisher-z). The lack of co-registered PE ⇔ ex vivo datasets prevents quantitative synthesis of spatial overlap or voxel-wise PET–metabolite correlations. Method papers demonstrate MSI ⇔ MRI registration that could be adapted to PET [16], and early HR-MAS studies already established micro-heterogeneity and 15 treatment effect markers. This gap motivates prospective hybrid PET/MR protocols with pre-registered stereotactic sampling, standardized spatial metrics (Dice/Jaccard; Fisher-z), and reporting templates, enabling downstream meta-analysis—aligned with current recommendations on PET accuracy and reproducibility [73,74,75,76,77,78].

### 4.8. Metabolic Guidance for Stereotactic Biopsy

In a single-center prospective series of non-enhancing gliomas, integrating ^18^F-FDOPA PET with MRI in the stereotactic workflow improved diagnostic yield (n = 20) [6]. Along with biopsy-controlled hybrid PET/MR studies, this supports amino acid PET-informed trajectories to mitigate sampling error in spatially heterogeneous tumors [18,19]. Regarding brain biopsies, we advocate for amino acid PET-informed trajectories (with hypoxia or receptor-targeted tracers when clinically indicated) integrated into neuronavigation, followed by standardized tissue banking for HR-MAS NMR spectroscopy and MALDI-MSI. This imaging-to-omics workflow is grounded in guideline-level recommendations for amino acid PET in gliomas and supported by biopsy-controlled hybrid PET/MR series [18,19,20,21]. Prospective studies should (i) pre-register PET/MR to the stereotactic space, (ii) spatially report concordance with Dice/Jaccard and voxel-wise correlations (Fisher-z), and (iii) share open protocols to enable reproducible meta-analysis and to reduce sampling error in spatially heterogeneous tumors.

### 4.9. Meta-Analytic Heterogeneity and Health-Economic Context

Pooled effects for OS were heterogeneous and non-significant under Hartung–Knapp-adjusted random-effects models, with I^2^ ≈82% for FDG and ≈88% for amino acid tracers and wide prediction intervals crossing the null. With only four studies per tracer class, meta-regression would have been statistically underpowered and unstable; we therefore did not attempt formal covariate modeling. Leave-one-out and influence analyses, including sensitivity analyses excluding dynamic FET metrics, did not materially alter pooled HRs or their wide prediction intervals, reinforcing the conclusion that baseline PET uptake has, at best, weak and context-dependent prognostic value for OS and does not support universal prognostic thresholds.

Consistent with this, EARL harmonization reduces inter-center PET variability and standardizes reconstruction [75]. From a payer perspective, amino- acid PET can be cost-effective: modeling for surgery planning and early temozolomide response suggests improved outcomes at acceptable or dominant costs [79,80]. Reimbursement remains uneven, underscoring the need for harmonized economic evidence. Trial standards increasingly emphasize clinical utility and reproducibility, including updated RANO 2.0 and proposed PET-based response frameworks [76].

### 4.10. Strengths and Limitations

Strengths include PROSPERO registration, duplicate screening, multi-tracer scope, and the first pooled synthesis integrating PET with metabolomics. Limitations include inter-scanner/reconstruction heterogeneity, variable ROI definitions, and a predominantly retrospective evidence base (58%). With <10 studies for some receptor-targeted tracers, small-study effects cannot be excluded; however, leave-one-out analyses yielded stable estimates, supporting robustness.

Second, we did not search Embase or Scopus because institutional access to these databases was not available. Although this may have led to the omission of some regional or gray literature, most high-impact neuro-oncology and nuclear medicine journals relevant to our questions are indexed in PubMed and Web of Science. Given our stringent eligibility criteria (HGG-only populations, predefined PET tracers, and the requirement for per-patient 2 × 2 data or HRs with 95% CIs), additional eligible studies from Embase/Scopus would be unlikely to materially alter the quantitative estimates, but some degree of database and publication bias cannot be excluded.

Thirdly, we did not formally assess small-study effects because no prognostic stratum included ≥10 studies; therefore, publication bias cannot be ruled out.

### 4.11. Clinical Implications

SUV/TBR plus a volumetric index (e.g., MTV or FET-BTV) has been reported at tumor boards, with genotype-aware interpretation per consensus [21,76]. Amino acid PET is integrated into surveillance—especially 3–6 months post-CRT, when MRI is confounded by PsP [21].

High-uptake subvolumes may guide dose painting, brachytherapy, or metabolism-directed drugs; LAT-1 inhibition is reported in first-in-human studies, and GLUT3 suppression radiosensitizes GBM preclinically [58,81]. Receptor-targeted tracers can triage for theranostics (CXCR4 and PSMA show feasibility and vascular/endothelial expression) [14,70].

### 4.12. Research Agenda to Guide Future Hybrid Imaging

(1) Align endpoints with RANO 2.0; predefine AA-PET algorithms for PsP/TRC; and report per-patient 2 × 2 tables and a single primary cutoff for pooling [76].
(2) Implement EARL-style harmonization (phantoms, reconstruction bands) and adhere to procedure standards to cut inter-center variance and enable credible multi-center HSROC [21,75].
(3) Prioritize within-patient head-to-head designs (e.g., FDOPA vs. FET) and controlled hybrid PET/MR workflows; for emerging targets (CXCR4, PSMA, FAPI), require per-patient diagnostic datasets with MRI comparators, extending early CXCR4 data for GBM vs. PCNSL [67,82].
(4) For radiomics/AI, mandate TRIPOD-AI reporting with external validation and code sharing; build IBSI-compliant features stress-tested on brain PET [74].
(5) Couple in vivo PET hotspots with ex vivo mapping (HR-MAS, MALDI-MSI) using pre-registered stereotactic sampling and standard spatial metrics (Dice/Jaccard; Fisher-z) to validate biopsy/dose painting/theranostic targets [21].
(6) Translate these elements into consensus policy and implementation frameworks to accelerate adoption and reimbursement of biologically coherent, multi-tracer hybrid imaging [76].

### 4.13. Implementation Barriers and Outreach

Adoption remains uneven across Europe; a 2024–2025 EORTC-BTG survey cites tracer availability, cost, and limited expertise as key barriers [83]. Targeted education and joint EANM–EANO workshops can disseminate acquisition standards and reporting templates to reduce variability [21]. Health-economic modeling suggests that amino acid PET is cost-effective for surgical planning/management, with downstream savings achieved by avoiding futile procedures [79,84]. Prospective metabolic biopsy series—e.g., FDOPA-guided stereotaxy in non-enhancing gliomas—support workflow integration and may help convince payers and guideline committees [6].

### 4.14. Research Roadmap

Priorities: multi-center trials with EARL-harmonized PET/MRI and central quality assurance [75]; radiomics/AI studies following TRIPOD-AI with external validation and public code [74]; evaluation of emerging tracers beyond glycolysis, with first-in-human data for ^18^F-FGln (glutamine) and ^18^F-FSPG (system x_c^−^/redox) [71,72].

Finally, integrative AI that fuses PET, MRI, genomics, metabolomics, and spatial transcriptomics could translate practice from histogram metrics to high-dimensional metabolic phenotyping and actionable risk scores [74].

## 5. Conclusions

Metabolic imaging offers a multidimensional view of glioma biology that could augment histomolecular classification; ex vivo techniques (HR-MAS NMR spectroscopy, MALDI-MSI) can cross-validate PET-derived signatures at the cellular scale, although current evidence lacks systematic spatial co-registration. In our synthesis, amino acid PET delivers the most consistent clinical value post-treatment, improving the discrimination of PsP and detection of recurrence when MRI is equivocal. The lack of voxel-wise PET–metabolite co-registration in human HGG remains the main barrier to quantitative imaging-to-omics concordance and should be a primary focus of future hybrid PET/MR protocols with stereotactic sampling. Baseline uptake metrics show weak, heterogeneous prognostic associations. We advocate for integrating AA-PET after chemoradiation uncertainty, reporting SUV/TBR and MTV/BTV with IDH-aware interpretation, and pursuing harmonized PET/MR-guided, tissue-validated studies.

## Figures and Tables

**Table 1:** PsP vs. progression—per-patient (HGG). Pooled rows are simple aggregations over study 2 × 2 totals; no model-based meta-analysis.

First Author (Year)	Tracer	Endpoint	N	TP	FP	FN	TN	Sensitivity	Specificity
**Pellerin** **(2021)** [23]	^18^F-FDOPA	PsP vs. progression (HGG, per-patient)	48	29	3	2	14	0.935	0.824
**Nabavizadeh (2023)** [24]	^18^F-FACBC	PsP vs. progression (HGG/GBM, per-patient)	28	21	1	1	5	0.955	0.833
**AA-PET pooled**	FDOPA + FACBC	PsP vs. progression (per-patient)	76	50	4	3	19	0.943	0.826

**Table 2:** Recurrence/progression vs. TRC—per-patient and HGG-only (main). The pooled FDOPA row is a simple aggregation over 2 × 2 totals. Per-lesion FET series are not tabulated. Legend—per-patient 2 × 2 counts for recurrence/progression versus treatment-related changes (TRC) in HGG (WHO III–IV); Se = TP/(TP + FN); Sp = TN/(TN + FP). Note: For Khangembam (2014) [27], FDG and 13N–NH3 have identical 2 × 2 counts in the HGG subset.

First Author (Year)	Tracer	Endpoint	N	TP	FP	FN	TN	Sensitivity	Specificity
**Herrmann (2014)** [25]	^18^F-FDOPA	Recurrence/progression vs. TRC (HGG, per-patient)	110	69	8	12	21	0.852	0.724
**Karunanithi (2013)** [26]	^18^F-FDOPA	Recurrence/progression vs. TRC (HGG, per-patient)	24	18	0	0	6	1.0	1.0
**Khangembam (2014)** [27]	^18^F-FDG	Recurrence/progression vs. TRC (HGG, per-patient)	18	7	3	2	6	0.778	0.667
**Khangembam (2014)** [27]	^13^N–NH_3_	Recurrence/progression vs. TRC (HGG, per-patient)	18	7	3	2	6	0.778	0.667
**FDOPA pooled**	^18^F-FDOPA	Recurrence/progression vs. TRC (HGG, per-patient)	134	87	8	12	27	0.879	0.771

## Data Availability

This meta-analysis used only study-level data extracted from published articles; no individual participant data were collected. The full database search strategies (Supplementary Table S1), the log of full-text exclusions (Supplementary Table S2), risk-of-bias assessments (Supplementary Tables S3 and S4), per-patient 2 × 2 diagnostic datasets (Supplementary Table S5), extracted hazard ratios with covariates (Supplementary Table S6), and the R code used for all meta-analyses (Supplementary Table S7) are provided as Supplementary Materials. PROSPERO: CRD420251113416.

## Supplementary Materials

The following supporting information can be downloaded at https://www.mdpi.com/article/10.3390/metabo16010017/s1: Table S1: Full database search strategies; Table S2: Full-text articles excluded at eligibility assessment; Table S3: QUADAS-2 (diagnostic accuracy); Table S4: QUIPS (prognosis: OS and PFS); Table S5: Per-patient 2 × 2 diagnostic datasets for pseudoprogression and recurrence/progression versus treatment-related change (with zero-cell flags); Table S6: Extracted hazard ratios, confidence intervals, and adjustment covariates for OS and PFS; Table S7: R code used for diagnostic and prognostic meta-analyses; Table S8: PRISMA 2020 checklist (main text and abstract).

## Abbreviations

AA-PET	Amino acid positron emission tomography
ATRX	Alpha thalassemia syndrome X-linked
AUC	Area under the receiver operating characteristic curve
BTV	Biological tumor volume
CRT	Chemoradiotherapy
CXCR4	C-X-C chemokine receptor type 4
DL	DerSimonian–Laird (random-effects model)
DSC-PWI	Dynamic susceptibility contrast perfusion-weighted imaging
EANM	European Association of Nuclear Medicine
EANO	European Association of Neuro-Oncology
EARL	EANM Research Ltd. (PET harmonization initiative)
EGFRvIII	Epidermal growth factor receptor variant III
FACBC	(^18^F-fluciclovine) Anti-1-amino-3-^18^F-fluorocyclobutane-1-carboxylic acid
FAZA	(^18^F-FAZA) Fluoroazomycin arabinoside PET tracer
FDG	(^18^F-FDG) 2-Deoxy-2-[^18^F]fluoro-D-glucose
FDOPA	(^18^F-FDOPA) 6-[^18^F]fluoro-L-3,4-dihydroxyphenylalanine
FET	(^18^F-FET) O-(2-[^18^F]fluoroethyl)-L-tyrosine
FGln	(^18^F-FGln) (2S,4R)-4-[^18^F]-fluoroglutamine
FMISO	(^18^F-FMISO) Fluoromisonidazole
FSPG	(^18^F-FSPG) 4-[^18^F]-fluoropropyl-L-glutamate (system x_c^−^ (xCT/SLC7A11))
GBM	Glioblastoma
HGG	High-grade glioma (WHO grade III–IV)
HK	Hartung–Knapp adjustment (random-effects)
HK2	Hexokinase-2
HR	Hazard ratio
HR-MAS	High-resolution magic-angle spinning (^1^H-NMR spectroscopy)
HSROC	Hierarchical summary receiver operating characteristic
IBSI	Image Biomarker Standardisation Initiative
IDH	Isocitrate dehydrogenase
IQR	Interquartile range
L/N ratio	Lesion-to-normal brain uptake ratio
LAT1	L-type amino acid transporter 1
MALDI-MSI	Matrix-assisted laser desorption/ionization mass-spectrometry imaging
MGMT	O6-methylguanine-DNA methyltransferase
MRI	Magnetic resonance imaging
MSI	Mass-spectrometry imaging
MTV	Metabolic tumor volume
nCBV	Normalized cerebral blood volume
OS	Overall survival
PET	Positron emission tomography
PET/CT	Positron emission tomography/computed tomography
PET/MR	Positron emission tomography/magnetic resonance
PFS	Progression-free survival
PI	Prediction interval
PSMA	Prostate-specific membrane antigen
PsP	Pseudoprogression
QUADAS-2	Quality Assessment of Diagnostic Accuracy Studies tool (version 2)
QUIPS	Quality in Prognosis Studies tool
RANO	Response Assessment in Neuro-Oncology
REML	Restricted maximum likelihood (random-effects estimator)
RN	Radiation necrosis
ROI	Region of interest
Se	Sensitivity
SNMMI	Society of Nuclear Medicine and Molecular Imaging
Sp	Specificity
SPECT	Single-photon emission computed tomography
SSTR	Somatostatin receptor
SUV	Standardized uptake value
SUVmax	Maximum standardized uptake value
TBR	Tumor-to-background ratio
TBRmean	Mean tumor-to-background ratio
TERT	Telomerase reverse transcriptase
TLG	Total lesion glycolysis
TRC	Treatment-related change(s)
TRIPOD-AI	Transparent Reporting of a multivariable prediction model for Individual Prognosis Or Diagnosis—Artificial Intelligence extension
TTP	Time-to-peak (dynamic PET)
WHO	World Health Organization

## References

[1] Miller K.D., Ostrom Q.T., Kruchko C., Patil N., Tihan T., Cioffi G., Fuchs H.E., Waite K.A., Jemal A., Siegel R.L. (2021). Brain and other central nervous system tumor statistics, 2021. CA Cancer J. Clin..

[2] Brandsma D., van den Bent M.J. (2009). Pseudoprogression and pseudoresponse in the treatment of gliomas. Curr. Opin. Neurol..

[3] Binneboese A., Covington M.F., Horn K.P., Archibald Z.G., Boucher K.M., Morton K.A., Hoffman J.M. (2021). Correlation between FDG-PET uptake and survival in patients with primary brain tumors. Am. J. Nucl. Med. Mol. Imaging.

[4] Tralins K.S., Douglas J.G., Stelzer K.J., Mankoff D.A., Silbergeld D.L., Rostomily R.C., Hummel S., Scharnhorst J., Krohn K.A., Spence A.M. (2002). Volumetric analysis of 18F-FDG PET in glioblastoma multiforme: Prognostic information and possible role in definition of target volumes in radiation dose escalation. J. Nucl. Med..

[5] Habermeier A., Graf J., Sandhofer B.F., Boissel J.P., Roesch F., Closs E.I. (2015). System L amino acid transporter LAT1 accumulates O-(2-fluoroethyl)-L-tyrosine (FET). Amino Acids.

[6] Todeschi J., Bund C., Cebula H., Chibbaro S., Lhermitte B., Pin Y., Lefebvre F., Namer I.J., Proust F. (2019). Diagnostic value of fusion of metabolic and structural images for stereotactic biopsy of brain tumors without enhancement after contrast medium injection. Neurochirurgie.

[7] Kunz M., Thon N., Eigenbrod S., Hartmann C., Egensperger R., Herms J., Geisler J., la Fougere C., Lutz J., Linn J. (2011). Hot spots in dynamic (18)FET-PET delineate malignant tumor parts within suspected WHO grade II gliomas. Neuro Oncol..

[8] Piroth M.D., Pinkawa M., Holy R., Klotz J., Nussen S., Stoffels G., Coenen H.H., Kaiser H.J., Langen K.J., Eble M.J. (2011). Prognostic value of early [18F]fluoroethyltyrosine positron emission tomography after radiochemotherapy in glioblastoma multiforme. Int. J. Radiat. Oncol. Biol. Phys..

[9] Mapelli P., Callea M., Fallanca F., Castellano A., Bailo M., Scifo P., Bettinardi V., Conte G.M., Monterisi C., Rancoita P.M.V. (2021). 18F-FAZA PET/CT in pretreatment assessment of hypoxic status in high-grade glioma: Correlation with hypoxia immunohistochemical biomarkers. Nucl. Med. Commun..

[10] Mapelli P., Picchio M. (2020). 18F-FAZA PET imaging in tumor hypoxia: A focus on high-grade glioma. Int. J. Biol. Markers.

[11] Gerstner E.R., Zhang Z., Fink J.R., Muzi M., Hanna L., Greco E., Prah M., Schmainda K.M., Mintz A., Kostakoglu L. (2016). ACRIN 6684: Assessment of Tumor Hypoxia in Newly Diagnosed Glioblastoma Using 18F-FMISO PET and MRI. Clin. Cancer Res..

[12] Lapa C., Luckerath K., Kleinlein I., Monoranu C.M., Linsenmann T., Kessler A.F., Rudelius M., Kropf S., Buck A.K., Ernestus R.I. (2016). (68)Ga-Pentixafor-PET/CT for Imaging of Chemokine Receptor 4 Expression in Glioblastoma. Theranostics.

[13] Verma P., Singh B.K., Sudhan M.D., Singh R.K., Bagul S.D., Chandak A.R., Soni B.K., Shelly D., Basu S. (2023). 68 Ga-PSMA-11 PET/CT Imaging in Brain Gliomas and Its Correlation With Clinicopathological Prognostic Parameters. Clin. Nucl. Med..

[14] Holzgreve A., Biczok A., Ruf V.C., Liesche-Starnecker F., Steiger K., Kirchner M.A., Unterrainer M., Mittlmeier L., Herms J., Schlegel J. (2021). PSMA Expression in Glioblastoma as a Basis for Theranostic Approaches: A Retrospective, Correlational Panel Study Including Immunohistochemistry, Clinical Parameters and PET Imaging. Front. Oncol..

[15] Cheng L.L., Anthony D.C., Comite A.R., Black P.M., Tzika A.A., Gonzalez R.G. (2000). Quantification of microheterogeneity in glioblastoma multiforme with ex vivo high-resolution magic-angle spinning (HRMAS) proton magnetic resonance spectroscopy. Neuro Oncol..

[16] Kampa J.M., Kellner U., Marsching C., Ramallo Guevara C., Knappe U.J., Sahin M., Giampa M., Niehaus K., Bednarz H. (2020). Glioblastoma multiforme: Metabolic differences to peritumoral tissue and IDH-mutated gliomas revealed by mass spectrometry imaging. Neuropathology.

[17] Elkhaled A., Jalbert L.E., Phillips J.J., Yoshihara H.A.I., Parvataneni R., Srinivasan R., Bourne G., Berger M.S., Chang S.M., Cha S. (2012). Magnetic resonance of 2-hydroxyglutarate in IDH1-mutated low-grade gliomas. Sci. Transl. Med..

[18] Song S., Cheng Y., Ma J., Wang L., Dong C., Wei Y., Xu G., An Y., Qi Z., Lin Q. (2020). Simultaneous FET-PET and contrast-enhanced MRI based on hybrid PET/MR improves delineation of tumor spatial biodistribution in gliomas: A biopsy validation study. Eur. J. Nucl. Med. Mol. Imaging.

[19] Mauler J., Lohmann P., Maudsley A.A., Sheriff S., Hoevels M., Meissner A.K., Hamisch C., Brunn A., Deckert M., Filss C.P. (2024). Diagnostic Accuracy of MR Spectroscopic Imaging and (18)F-FET PET for Identifying Glioma: A Biopsy-Controlled Hybrid PET/MRI Study. J. Nucl. Med..

[20] Galldiks N., Lohmann P., Aboian M., Barajas R.F., Breen W.G., Ivanidze J., Johnson D.R., Kaufmann T.J., Kim M.M., Mair M.J. (2025). Update to the RANO working group and EANO recommendations for the clinical use of PET imaging in gliomas. Lancet Oncol..

[21] Law I., Albert N.L., Arbizu J., Boellaard R., Drzezga A., Galldiks N., la Fougere C., Langen K.J., Lopci E., Lowe V. (2019). Joint EANM/EANO/RANO practice guidelines/SNMMI procedure standards for imaging of gliomas using PET with radiolabelled amino acids and [(18)F]FDG: Version 1.0. Eur. J. Nucl. Med. Mol. Imaging.

[22] Shahzadi I., Seidlitz A., Beuthien-Baumann B., Zwanenburg A., Platzek I., Kotzerke J., Baumann M., Krause M., Troost E.G.C., Lock S. (2024). Radiomics for residual tumour detection and prognosis in newly diagnosed glioblastoma based on postoperative [(11)C] methionine PET and T1c-w MRI. Sci. Rep..

[23] Pellerin A., Khalife M., Sanson M., Rozenblum-Beddok L., Bertaux M., Soret M., Galanaud D., Dormont D., Kas A., Pyatigorskaya N. (2021). Simultaneously acquired PET and ASL imaging biomarkers may be helpful in differentiating progression from pseudo-progression in treated gliomas. Eur. Radiol..

[24] Nabavizadeh A., Bagley S.J., Doot R.K., Ware J.B., Young A.J., Ghodasara S., Zhao C., Anderson H., Schubert E., Carpenter E.L. (2023). Distinguishing Progression from Pseudoprogression in Glioblastoma Using (18)F-Fluciclovine PET. J. Nucl. Med..

[25] Herrmann K., Czernin J., Cloughesy T., Lai A., Pomykala K.L., Benz M.R., Buck A.K., Phelps M.E., Chen W. (2014). Comparison of visual and semiquantitative analysis of 18F-FDOPA-PET/CT for recurrence detection in glioblastoma patients. Neuro Oncol..

[26] Karunanithi S., Sharma P., Kumar A., Khangembam B.C., Bandopadhyaya G.P., Kumar R., Goenka A., Gupta D.K., Malhotra A., Bal C. (2013). Comparative diagnostic accuracy of contrast-enhanced MRI and (18)F-FDOPA PET-CT in recurrent glioma. Eur. Radiol..

[27] Khangembam B.C., Karunanithi S., Sharma P., Kc S.S., Kumar R., Julka P.K., Kumar R., Bal C. (2014). Perfusion-metabolism coupling in recurrent gliomas: A prospective validation study with 13N-ammonia and 18F-fluorodeoxyglucose PET/CT. Neuroradiology.

[28] Jansen N.L., Suchorska B., Wenter V., Schmid-Tannwald C., Todica A., Eigenbrod S., Niyazi M., Tonn J.C., Bartenstein P., Kreth F.W. (2015). Prognostic significance of dynamic 18F-FET PET in newly diagnosed astrocytic high-grade glioma. J. Nucl. Med..

[29] Rozenblum L., Zaragori T., Tran S., Morales-Martinez A., Taillandier L., Blonski M., Rech F., Galanaud D., Kas A., Verger A. (2023). Differentiating high-grade glioma progression from treatment-related changes with dynamic [(18)F]FDOPA PET: A multicentric study. Eur. Radiol..

[30] Miller S., Li P., Schipper M., Junck L., Piert M., Lawrence T.S., Tsien C., Cao Y., Kim M.M. (2020). Metabolic Tumor Volume Response Assessment Using (11)C-Methionine Positron Emission Tomography Identifies Glioblastoma Tumor Subregions That Predict Progression Better Than Baseline or Anatomic Magnetic Resonance Imaging Alone. Adv. Radiat. Oncol..

[31] Huang S., Michalek J.E., Reardon D.A., Wen P.Y., Floyd J.R., Fox P.T., Clarke G.D., Jerabek P.A., Schmainda K.M., Muzi M. (2021). Assessment of tumor hypoxia and perfusion in recurrent glioblastoma following bevacizumab failure using MRI and (18)F-FMISO PET. Sci. Rep..

[32] Colavolpe C., Chinot O., Metellus P., Mancini J., Barrie M., Bequet-Boucard C., Tabouret E., Mundler O., Figarella-Branger D., Guedj E. (2012). FDG-PET predicts survival in recurrent high-grade gliomas treated with bevacizumab and irinotecan. Neuro Oncol..

[33] Leiva-Salinas C., Schiff D., Flors L., Patrie J.T., Rehm P.K. (2017). FDG PET/MR Imaging Coregistration Helps Predict Survival in Patients with Glioblastoma and Radiologic Progression after Standard of Care Treatment. Radiology.

[34] Chiang G.C., Galla N., Ferraro R., Kovanlikaya I. (2017). The Added Prognostic Value of Metabolic Tumor Size on FDG-PET at First Suspected Recurrence of Glioblastoma Multiforme. J. Neuroimaging.

[35] Graham M.S., Krebs S., Bale T., Domfe K., Lobaugh S.M., Zhang Z., Dunphy M.P., Kaley T., Young R.J. (2020). Value of [(18)F]-FDG positron emission tomography in patients with recurrent glioblastoma receiving bevacizumab. Neurooncol Adv..

[36] Suchorska B., Jansen N.L., Linn J., Kretzschmar H., Janssen H., Eigenbrod S., Simon M., Popperl G., Kreth F.W., la Fougere C. (2015). Biological tumor volume in 18FET-PET before radiochemotherapy correlates with survival in GBM. Neurology.

[37] Bauer E.K., Stoffels G., Blau T., Reifenberger G., Felsberg J., Werner J.M., Lohmann P., Rosen J., Ceccon G., Tscherpel C. (2020). Prediction of survival in patients with IDH-wildtype astrocytic gliomas using dynamic O-(2-[(18)F]-fluoroethyl)-L-tyrosine PET. Eur. J. Nucl. Med. Mol. Imaging.

[38] Wirsching H.G., Roelcke U., Weller J., Hundsberger T., Hottinger A.F., von Moos R., Caparrotti F., Conen K., Remonda L., Roth P. (2021). MRI and (18)FET-PET Predict Survival Benefit from Bevacizumab Plus Radiotherapy in Patients with Isocitrate Dehydrogenase Wild-type Glioblastoma: Results from the Randomized ARTE Trial. Clin. Cancer Res..

[39] Zhao F., Cui Y., Li M., Fu Z., Chen Z., Kong L., Yang G., Yu J. (2014). Prognostic value of 3′-deoxy-3′-18F-fluorothymidine ([(18)F] FLT PET) in patients with recurrent malignant gliomas. Nucl. Med. Biol..

[40] Bai J., Cui B., Li F., Han X., Yang H., Lu J. (2025). Multiparametric radiomics signature for predicting molecular genotypes in adult-type diffuse gliomas utilizing (18)F-FET PET/MRI. BMC Med. Imaging.

[41] Kaiser L., Quach S., Zounek A.J., Wiestler B., Zatcepin A., Holzgreve A., Bollenbacher A., Bartos L.M., Ruf V.C., Boning G. (2024). Enhancing predictability of IDH mutation status in glioma patients at initial diagnosis: A comparative analysis of radiomics from MRI, [(18)F]FET PET, and TSPO PET. Eur. J. Nucl. Med. Mol. Imaging.

[42] Lohmeier J., Radbruch H., Brenner W., Hamm B., Tietze A., Makowski M.R. (2023). Predictive IDH Genotyping Based on the Evaluation of Spatial Metabolic Heterogeneity by Compartmental Uptake Characteristics in Preoperative Glioma Using (18)F-FET PET. J. Nucl. Med..

[43] Zaragori T., Oster J., Roch V., Hossu G., Chawki M.B., Grignon R., Pouget C., Gauchotte G., Rech F., Blonski M. (2022). (18)F-FDOPA PET for the Noninvasive Prediction of Glioma Molecular Parameters: A Radiomics Study. J. Nucl. Med..

[44] Zaragori T., Doyen M., Rech F., Blonski M., Taillandier L., Imbert L., Verger A. (2021). Dynamic (18)F-FDopa PET Imaging for Newly Diagnosed Gliomas: Is a Semiquantitative Model Sufficient?. Front. Oncol..

[45] Zhou W., Wen J., Huang Q., Zeng Y., Zhou Z., Zhu Y., Chen L., Guan Y., Xie F., Zhuang D. (2024). Development and validation of clinical-radiomics analysis for preoperative prediction of IDH mutation status and WHO grade in diffuse gliomas: A consecutive L-[methyl-11C] methionine cohort study with two PET scanners. Eur. J. Nucl. Med. Mol. Imaging.

[46] Nakajo K., Uda T., Kawashima T., Terakawa Y., Ishibashi K., Tsuyuguchi N., Tanoue Y., Nagahama A., Uda H., Koh S. (2021). Diagnostic Performance of [(11)C]Methionine Positron Emission Tomography in Newly Diagnosed and Untreated Glioma Based on the Revised World Health Organization 2016 Classification. World Neurosurg..

[47] Song S., Wang L., Yang H., Shan Y., Cheng Y., Xu L., Dong C., Zhao G., Lu J. (2021). Static (18)F-FET PET and DSC-PWI based on hybrid PET/MR for the prediction of gliomas defined by IDH and 1p/19q status. Eur. Radiol..

[48] Lan C., Li H., Wang L., Zhang J., Wang X., Zhang R., Yuan X., Wu T., Wu J., Lu M. (2021). Absolute quantification of 2-hydroxyglutarate on tissue by matrix-assisted laser desorption/ionization mass spectrometry imaging for rapid and precise identification of isocitrate dehydrogenase mutations in human glioma. Int. J. Cancer.

[49] Chen W., Lou H., Zhang H., Nie X., Lan W., Yang Y., Xiang Y., Qi J., Lei H., Tang H. (2011). Grade classification of neuroepithelial tumors using high-resolution magic-angle spinning proton nuclear magnetic resonance spectroscopy and pattern recognition. Sci. China Life Sci..

[50] Longuespee R., Wefers A.K., De Vita E., Miller A.K., Reuss D.E., Wick W., Herold-Mende C., Kriegsmann M., Schirmacher P., von Deimling A. (2018). Rapid detection of 2-hydroxyglutarate in frozen sections of IDH mutant tumors by MALDI-TOF mass spectrometry. Acta Neuropathol. Commun..

[51] Poulsen S.H., Urup T., Grunnet K., Christensen I.J., Larsen V.A., Jensen M.L., Af Rosenschold P.M., Poulsen H.S., Law I. (2017). The prognostic value of FET PET at radiotherapy planning in newly diagnosed glioblastoma. Eur. J. Nucl. Med. Mol. Imaging.

[52] Rosen J., Stoffels G., Lohmann P., Bauer E.K., Werner J.M., Wollring M., Rapp M., Felsberg J., Kocher M., Fink G.R. (2021). Prognostic value of pre-irradiation FET PET in patients with not completely resectable IDH-wildtype glioma and minimal or absent contrast enhancement. Sci. Rep..

[53] Zhang Q., Gao X., Wei G., Qiu C., Qu H., Zhou X. (2019). Prognostic Value of MTV, SUVmax and the T/N Ratio of PET/CT in Patients with Glioma: A Systematic Review and Meta-Analysis. J. Cancer.

[54] Mauler J., Maudsley A.A., Langen K.J., Nikoubashman O., Stoffels G., Sheriff S., Lohmann P., Filss C., Galldiks N., Kops E.R. (2018). Spatial Relationship of Glioma Volume Derived from (18)F-FET PET and Volumetric MR Spectroscopy Imaging: A Hybrid PET/MRI Study. J. Nucl. Med..

[55] Gandia-Gonzalez M.L., Cerdan S., Barrios L., Lopez-Larrubia P., Feijoo P.G., Palpan A., Roda J.M., Solivera J. (2019). Assessment of Overall Survival in Glioma Patients as Predicted by Metabolomic Criteria. Front. Oncol..

[56] Liu H., Liu N., Cheng Y., Jin W., Zhang P., Wang X., Yang H., Xu X., Wang Z., Tu Y. (2017). Hexokinase 2 (HK2), the tumor promoter in glioma, is downregulated by miR-218/Bmi1 pathway. PLoS ONE.

[57] Vartanian A., Agnihotri S., Wilson M.R., Burrell K.E., Tonge P.D., Alamsahebpour A., Jalali S., Taccone M.S., Mansouri S., Golbourn B. (2016). Targeting hexokinase 2 enhances response to radio-chemotherapy in glioblastoma. Oncotarget.

[58] Pucci G., Minafra L., Bravata V., Calvaruso M., Turturici G., Cammarata F.P., Savoca G., Abbate B., Russo G., Cavalieri V. (2024). Glut-3 Gene Knockdown as a Potential Strategy to Overcome Glioblastoma Radioresistance. Int. J. Mol. Sci..

[59] Liu X., Cao Z., Wang W., Zou C., Wang Y., Pan L., Jia B., Zhang K., Zhang W., Li W. (2023). Engineered Extracellular Vesicle-Delivered CRISPR/Cas9 for Radiotherapy Sensitization of Glioblastoma. ACS Nano.

[60] Zhu G.D., Yu J., Sun Z.Y., Chen Y., Zheng H.M., Lin M.L., Ou-Yang S., Liu G.L., Zhang J.W., Shao F.M. (2021). Genome-wide CRISPR/Cas9 screening identifies CARHSP1 responsible for radiation resistance in glioblastoma. Cell Death Dis..

[61] Dev I.D., Puranik A.D., Purandare N.C., Gupta T., Sridhar E., Shetty P., Moiyadi A., Agrawal A., Shah S., Rangarajan V. (2021). Prognostic significance of 18F-FDG PET/CT parameters in IDH-1 wild-type GBM and correlation with molecular markers. Nucl. Med. Commun..

[62] Izquierdo-Garcia J.L., Viswanath P., Eriksson P., Cai L., Radoul M., Chaumeil M.M., Blough M., Luchman H.A., Weiss S., Cairncross J.G. (2015). IDH1 Mutation Induces Reprogramming of Pyruvate Metabolism. Cancer Res..

[63] Masui K., Tanaka K., Ikegami S., Villa G.R., Yang H., Yong W.H., Cloughesy T.F., Yamagata K., Arai N., Cavenee W.K. (2015). Glucose-dependent acetylation of Rictor promotes targeted cancer therapy resistance. Proc. Natl. Acad. Sci. USA.

[64] Mashimo T., Pichumani K., Vemireddy V., Hatanpaa K.J., Singh D.K., Sirasanagandla S., Nannepaga S., Piccirillo S.G., Kovacs Z., Foong C. (2014). Acetate is a bioenergetic substrate for human glioblastoma and brain metastases. Cell.

[65] Ahrari S., Zaragori T., Bros M., Oster J., Imbert L., Verger A. (2022). Implementing the Point Spread Function Deconvolution for Better Molecular Characterization of Newly Diagnosed Gliomas: A Dynamic (18)F-FDOPA PET Radiomics Study. Cancers.

[66] Ouyang Z.Q., Zheng G.R., Duan X.R., Zhang X.R., Ke T.F., Bao S.S., Yang J., He B., Liao C.D. (2023). Diagnostic accuracy of glioma pseudoprogression identification with positron emission tomography imaging: A systematic review and meta-analysis. Quant. Imaging Med. Surg..

[67] Yu P., Wang Y., Su F., Chen Y. (2023). Comparing [18F]FET PET and [18F]FDOPA PET for glioma recurrence diagnosis: A systematic review and meta-analysis. Front. Oncol..

[68] Lo Greco M.C., Milazzotto R., Liardo R.L.E., Acquaviva G., La Rocca M., Altieri R., Certo F., Barbagallo G.M., Basile A., Foti P.V. (2022). Relapsing High-Grade Glioma from Peritumoral Zone: Critical Review of Radiotherapy Treatment Options. Brain Sci..

[69] Zwanenburg A., Vallieres M., Abdalah M.A., Aerts H., Andrearczyk V., Apte A., Ashrafinia S., Bakas S., Beukinga R.J., Boellaard R. (2020). The Image Biomarker Standardization Initiative: Standardized Quantitative Radiomics for High-Throughput Image-based Phenotyping. Radiology.

[70] Jacobs S.M., Wesseling P., de Keizer B., Tolboom N., Ververs F.F.T., Krijger G.C., Westerman B.A., Snijders T.J., Robe P.A., van der Kolk A.G. (2022). CXCR4 expression in glioblastoma tissue and the potential for PET imaging and treatment with [(68)Ga]Ga-Pentixafor /[(177)Lu]Lu-Pentixather. Eur. J. Nucl. Med. Mol. Imaging.

[71] Dunphy M.P.S., Harding J.J., Venneti S., Zhang H., Burnazi E.M., Bromberg J., Omuro A.M., Hsieh J.J., Mellinghoff I.K., Staton K. (2018). In Vivo PET Assay of Tumor Glutamine Flux and Metabolism: In-Human Trial of (18)F-(2S,4R)-4-Fluoroglutamine. Radiology.

[72] Wardak M., Sonni I., Fan A.P., Minamimoto R., Jamali M., Hatami N., Zaharchuk G., Fischbein N., Nagpal S., Li G. (2022). (18)F-FSPG PET/CT Imaging of System x(C)(-) Transporter Activity in Patients with Primary and Metastatic Brain Tumors. Radiology.

[73] de Zwart P.L., van Dijken B.R.J., Holtman G.A., Stormezand G.N., Dierckx R., Jan van Laar P., van der Hoorn A. (2020). Diagnostic Accuracy of PET Tracers for the Differentiation of Tumor Progression from Treatment-Related Changes in High-Grade Glioma: A Systematic Review and Metaanalysis. J. Nucl. Med..

[74] Collins G.S., Moons K.G.M., Dhiman P., Riley R.D., Beam A.L., Van Calster B., Ghassemi M., Liu X., Reitsma J.B., van Smeden M. (2024). TRIPOD+AI statement: Updated guidance for reporting clinical prediction models that use regression or machine learning methods. BMJ.

[75] Aide N., Lasnon C., Veit-Haibach P., Sera T., Sattler B., Boellaard R. (2017). EANM/EARL harmonization strategies in PET quantification: From daily practice to multicentre oncological studies. Eur. J. Nucl. Med. Mol. Imaging.

[76] Wen P.Y., van den Bent M., Youssef G., Cloughesy T.F., Ellingson B.M., Weller M., Galanis E., Barboriak D.P., de Groot J., Gilbert M.R. (2023). RANO 2.0: Update to the Response Assessment in Neuro-Oncology Criteria for High- and Low-Grade Gliomas in Adults. J. Clin. Oncol..

[77] Albert N.L., Galldiks N., Ellingson B.M., van den Bent M.J., Chang S.M., Cicone F., de Groot J., Koh E.S., Law I., Le Rhun E. (2024). PET-based response assessment criteria for diffuse gliomas (PET RANO 1.0): A report of the RANO group. Lancet Oncol..

[78] Azimi M.S., Cheraghi M., MahdiMaleki F., MahdiMaleki F., Sanaat A., Hoilund-Carlsen P.F., Alavi A., Zaidi H. (2025). Toward standardization: Assessing the reproducibility of radiomics features in partial volume-corrected brain PET images. Neuroimage.

[79] Heinzel A., Stock S., Langen K.J., Muller D. (2012). Cost-effectiveness analysis of amino acid PET-guided surgery for supratentorial high-grade gliomas. J. Nucl. Med..

[80] Rosen J., Ceccon G., Bauer E.K., Werner J.M., Tscherpel C., Dunkl V., Rapp M., Sabel M., Herrlinger U., Heinzel A. (2022). Cost Effectiveness of (18)F-FET PET for Early Treatment Response Assessment in Glioma Patients After Adjuvant Temozolomide Chemotherapy. J. Nucl. Med..

[81] Okano N., Naruge D., Kawai K., Kobayashi T., Nagashima F., Endou H., Furuse J. (2020). First-in-human phase I study of JPH203, an L-type amino acid transporter 1 inhibitor, in patients with advanced solid tumors. Investig. New Drugs.

[82] Chen Z., Liu H., Yang A., Liao J., Wu Z., Chen J., Miao W. (2025). 68 Ga-Pentixafor PET in Combination With MRI Improves the Differential Diagnosis of Glioblastoma and Primary Central Nervous System Lymphoma. Clin. Nucl. Med..

[83] Mair M.J., Lohmann P., Galldiks N., Belting M., Brandal P., Broen M.P.G., Cicone F., Daisne J.F., Ducray F., Ehret F. (2025). Availability and use of PET in patients with brain tumours—A European Organisation for Research and Treatment of Cancer—Brain Tumour Group (EORTC-BTG) survey. Eur. J. Nucl. Med. Mol. Imaging.

[84] Heinzel A., Stock S., Langen K.J., Muller D. (2012). Cost-effectiveness analysis of FET PET-guided target selection for the diagnosis of gliomas. Eur. J. Nucl. Med. Mol. Imaging.

