# Supplementary material for: Metabolic Heterogeneity in High-Grade Glioma Assessed by Multi-Tracer PET and Ex Vivo Metabolomics: A Systematic Review and Meta-Analysis
Source: Metabolites. 2025 Dec 24;16(1):17. doi: 10.3390/metabo16010017 (PMC12844024; doi:10.3390/metabo16010017)
Supplement: Supplementary file 1 [file metabolites-16-00017-s001.zip › Table S1. Full database search .docx]

**Supplementary Table S1. Full database search strategies**

PRISMA item 7 (Search strategy). Date of last search: 1 September 2025.

**Database: PubMed/MEDLINE (NCBI)**

Limits/filters: humans-only (animal-only records excluded), no language restriction, publication date from inception to 2025-09-01.

Exact query :

("Glioma"[Mesh] OR glioma*[tiab] OR glioblastom*[tiab] OR "high-grade glioma"[tiab] OR HGG[tiab] OR astrocytoma*[tiab] OR oligodendroglioma*[tiab])
 AND
 ("Positron-Emission Tomography"[Mesh] OR "positron emission tomograph*"[tiab] OR PET[tiab]
 OR FDG[tiab] OR fluorodeoxyglucose[tiab]
 OR FMISO[tiab] OR fluoromisonidazole[tiab] OR FAZA[tiab] OR HX4[tiab] OR "64Cu-ATSM"[tiab]
 OR FET[tiab] OR "fluoroethyltyrosine"[tiab] OR "O-(2-[18F]-fluoroethyl)-L-tyrosine"[tiab]
 OR FDOPA[tiab] OR "F-DOPA"[tiab] OR "6-[18F]-fluoro-L-dopa"[tiab]
 OR MET[tiab] OR "11C-methionine"[tiab] OR methionine[tiab] OR FAMT[tiab]
 OR "18F-FSPG"[tiab] OR FSPG[tiab] OR "BAY 94-9392"[tiab]
 OR TSPO[tiab] OR DOTATATE[tiab] OR PSMA[tiab])
 AND
 (pseudoprogress*[tiab] OR "pseudo progression"[tiab]
 OR "treatment-related change*"[tiab] OR "treatment related change*"[tiab] OR "radiation necrosis"[tiab]
 OR recurren*[tiab] OR progres*[tiab]
 OR "overall survival"[tiab] OR "progression-free survival"[tiab] OR OS[tiab] OR PFS[tiab]
 OR IDH[tiab] OR "isocitrate dehydrogenase"[tiab] OR MGMT[tiab] OR "1p/19q"[tiab] OR ATRX[tiab] OR TERT[tiab] OR "molecular marker*"[tiab]
 OR stereotactic[tiab] OR biopsy[tiab]
 OR "HR-MAS"[tiab] OR HRMAS[tiab] OR "high-resolution magic angle spinning"[tiab]
 OR "MALDI-MSI"[tiab] OR "MALDI MSI"[tiab] OR "MALDI imaging"[tiab] OR "mass spectrometry imaging"[tiab] )
AND ("1900/01/01"[Date - Publication] : "2025/09/01"[Date - Publication])
NOT (animals[mh] NOT humans[mh])

**Database: Web of Science Core Collection (Clarivate)**

Indexes: SCI-EXPANDED, SSCI, A&HCI, ESCI (Core Collection).

Limits/filters: no language restriction; document types: Article OR Review; timespan: inception–2025-09-01.

Exact query (Advanced Search):

TS=(
 (glioma* OR glioblastom* OR "high-grade glioma" OR HGG OR astrocytoma* OR oligodendroglioma*)
 AND
 ("positron emission tomograph*" OR PET OR FDG OR fluorodeoxyglucose
 OR FMISO OR fluoromisonidazole OR FAZA OR HX4 OR "64Cu-ATSM"
 OR FET OR fluoroethyltyrosine OR "O-(2-[18F]-fluoroethyl)-L-tyrosine"
 OR FDOPA OR "F-DOPA" OR "6-[18F]-fluoro-L-dopa"
 OR MET OR "11C-methionine" OR methionine OR FAMT
 OR "18F-FSPG" OR FSPG OR "BAY 94-9392"
 OR TSPO OR DOTATATE OR PSMA )
 AND
 (pseudoprogress* OR "pseudo progression"
 OR "treatment-related change*" OR "treatment related change*" OR "radiation necrosis"
 OR recurren* OR progres*
 OR "overall survival" OR "progression-free survival" OR OS OR PFS
 OR IDH OR "isocitrate dehydrogenase" OR MGMT OR "1p/19q" OR ATRX OR TERT OR "molecular marker*"
 OR stereotactic OR biopsy
 OR "HR-MAS" OR HRMAS OR "high-resolution magic angle spinning"
 OR "MALDI-MSI" OR "MALDI MSI" OR "MALDI imaging" OR "mass spectrometry imaging" )
Refined by: DOCUMENT TYPES=(ARTICLE OR REVIEW)
Timespan=1900-2025; Search date=2025-09-01

**Reproducibility notes**

• No language limits were applied at the search stage; non-English records were screened.

• PubMed humans-only implemented via NOT (animals[mh] NOT humans[mh]).

• Deduplication performed after export (EndNote).

• All records retrieved up to 2025-09-01 (inclusive).
