# Supplementary material for: Metabolic Heterogeneity in High-Grade Glioma Assessed by Multi-Tracer PET and Ex Vivo Metabolomics: A Systematic Review and Meta-Analysis
Source: Metabolites. 2025 Dec 24;16(1):17. doi: 10.3390/metabo16010017 (PMC12844024; doi:10.3390/metabo16010017)
Supplement: Supplementary file 1 [file metabolites-16-00017-s001.zip › Table S2. Full鈥憈ext articles excluded .docx]

| First author | Year | Primary reason | Notes |
| --- | --- | --- | --- |
| Bai[40] | 2025 | No extractable 2×2 or HR (ROC/AUC or qualitative only) | Reported ROC/AUC or qualitative results only; no per‑patient 2×2 or HR with 95% CI available. |
| Carles [85] | 2021 | No extractable 2×2 or HR (ROC/AUC or qualitative only) | Reported ROC/AUC or qualitative results only; no per‑patient 2×2 or HR with 95% CI available. |
| Castello [86] | 2020 | Not HGG-only or mixed-grade not separable | Population mixed or not high‑grade‑only; HGG subset not separable for extraction. |
| Ceccon [87] | 2021 | Interventional PET-guided study (not eligible) | Interventional PET‑guided design (monitoring or PET‑guided resection/therapy); not eligible per protocol. |
| Chen [89] | 2005 | Not HGG-only or mixed-grade not separable | Population mixed or not high‑grade‑only; HGG subset not separable for extraction. |
| Chen [88] | 2023 | Non-original (review/editorial/abstract) | Non‑original article (review/editorial/abstract); not eligible. |
| Chen [82] | 2025 | No extractable 2×2 or HR (ROC/AUC or qualitative only) | Reported ROC/AUC or qualitative results only; no per‑patient 2×2 or HR with 95% CI available. |
| Clément [90] | 2022 | No extractable 2×2 or HR (ROC/AUC or qualitative only) | Reported ROC/AUC or qualitative results only; no per‑patient 2×2 or HR with 95% CI available. |
| Cui [91] | 2021 | Not HGG-only or mixed-grade not separable | Population mixed or not high‑grade‑only; HGG subset not separable for extraction. |
| Dankbaar [93] | 2015 | No extractable 2×2 or HR (ROC/AUC or qualitative only) | Reported ROC/AUC or qualitative results only; no per‑patient 2×2 or HR with 95% CI available. |
| Debreczeni-Máté [94] | 2024 | No extractable 2×2 or HR (ROC/AUC or qualitative only) | Reported ROC/AUC or qualitative results only; no per‑patient 2×2 or HR with 95% CI available. |
| Dissaux [95] | 2020 | Interventional PET-guided study (not eligible) | Interventional PET‑guided design (monitoring or PET‑guided resection/therapy); not eligible per protocol. |
| D’Souza [92] | 2014 | Not HGG-only or mixed-grade not separable | Population mixed or not high‑grade‑only; HGG subset not separable for extraction. |
| Enslow [96] | 2012 | Not HGG-only or mixed-grade not separable | Population mixed or not high‑grade‑only; HGG subset not separable for extraction. |
| Galldiks [97] | 2015 | No extractable 2×2 or HR (ROC/AUC or qualitative only) | Reported ROC/AUC or qualitative results only; no per‑patient 2×2 or HR with 95% CI available. |
| Garcia [98] | 2017 | Not HGG-only or mixed-grade not separable | Population mixed or not high‑grade‑only; HGG subset not separable for extraction. |
| Hajri [99] | 2023 | No extractable 2×2 or HR (ROC/AUC or qualitative only) | Reported ROC/AUC or qualitative results only; no per‑patient 2×2 or HR with 95% CI available. |
| Harris [100] | 2012 | No extractable 2×2 or HR (ROC/AUC or qualitative only) | Reported ROC/AUC or qualitative results only; no per‑patient 2×2 or HR with 95% CI available. |
| Hojjati [101] | 2018 | No extractable 2×2 or HR (ROC/AUC or qualitative only) | Reported ROC/AUC or qualitative results only; no per‑patient 2×2 or HR with 95% CI available. |
| Imani [102] | 2012 | Not HGG-only or mixed-grade not separable | Population mixed or not high‑grade‑only; HGG subset not separable for extraction. |
| Jeong [103] | 2010 | Not HGG-only or mixed-grade not separable | Population mixed or not high‑grade‑only; HGG subset not separable for extraction. |
| Kaiser [41] | 2024 | No extractable 2×2 or HR (ROC/AUC or qualitative only) | Reported ROC/AUC or qualitative results only; no per‑patient 2×2 or HR with 95% CI available. |
| Karlberg [104] | 2024 | No extractable 2×2 or HR (ROC/AUC or qualitative only) | Reported ROC/AUC or qualitative results only; no per‑patient 2×2 or HR with 95% CI available. |
| Kebir [105] | 2016 | No extractable 2×2 or HR (ROC/AUC or qualitative only) | Reported ROC/AUC or qualitative results only; no per‑patient 2×2 or HR with 95% CI available. |
| Kobayashi [106] | 2015 | No extractable 2×2 or HR (ROC/AUC or qualitative only) | Reported ROC/AUC or qualitative results only; no per‑patient 2×2 or HR with 95% CI available. |
| Lapa [107] | 2014 | No extractable 2×2 or HR (ROC/AUC or qualitative only) | Reported ROC/AUC or qualitative results only; no per‑patient 2×2 or HR with 95% CI available. |
| Li [108] | 2021 | No extractable 2×2 or HR (ROC/AUC or qualitative only) | Reported ROC/AUC or qualitative results only; no per‑patient 2×2 or HR with 95% CI available. |
| Liu [109] | 2021 | No extractable 2×2 or HR (ROC/AUC or qualitative only) | Reported ROC/AUC or qualitative results only; no per‑patient 2×2 or HR with 95% CI available. |
| Lohmeier [42] | 2023 | No extractable 2×2 or HR (ROC/AUC or qualitative only) | Reported ROC/AUC or qualitative results only; no per‑patient 2×2 or HR with 95% CI available. |
| Lohmeier [110] | 2024 | No extractable 2×2 or HR (ROC/AUC or qualitative only) | Reported ROC/AUC or qualitative results only; no per‑patient 2×2 or HR with 95% CI available. |
| Mattoli[111] | 2021 | Not HGG-only or mixed-grade not separable | Population mixed or not high‑grade‑only; HGG subset not separable for extraction. |
| Møller [112] | 2016 | Interventional PET-guided study (not eligible) | Interventional PET‑guided design (monitoring or PET‑guided resection/therapy); not eligible per protocol. |
| Nakajo [46] | 2021 | No extractable 2×2 or HR (ROC/AUC or qualitative only) | Reported ROC/AUC or qualitative results only; no per‑patient 2×2 or HR with 95% CI available. |
| Norikane [113] | 2024 | No extractable 2×2 or HR (ROC/AUC or qualitative only) | Reported ROC/AUC or qualitative results only; no per‑patient 2×2 or HR with 95% CI available. |
| Ort [114] | 2024 | Interventional PET-guided study (not eligible) | Interventional PET‑guided design (monitoring or PET‑guided resection/therapy); not eligible per protocol. |
| Oster [115] | 2024 | No extractable 2×2 or HR (ROC/AUC or qualitative only) | Reported ROC/AUC or qualitative results only; no per‑patient 2×2 or HR with 95% CI available. |
| Pardo [116] | 2004 | No extractable 2×2 or HR (ROC/AUC or qualitative only) | Reported ROC/AUC or qualitative results only; no per‑patient 2×2 or HR with 95% CI available. |
| Park [117] | 2018 | No extractable 2×2 or HR (ROC/AUC or qualitative only) | Reported ROC/AUC or qualitative results only; no per‑patient 2×2 or HR with 95% CI available. |
| Patronas [118] | 1985 | Not eligible per protocol | Not eligible per prespecified protocol criteria. |
| Pełka [119] | 2025 | Not eligible per protocol | Not eligible per prespecified protocol criteria. |
| Poulsen [120] | 2017 | Not eligible per protocol | Not eligible per prespecified protocol criteria. |
| Qiao [121] | 2019 | No extractable 2×2 or HR (ROC/AUC or qualitative only) | Reported ROC/AUC or qualitative results only; no per‑patient 2×2 or HR with 95% CI available. |
| Roach [122] | 2022 | Not eligible per protocol | Not eligible per prespecified protocol criteria. |
| Robert [123] | 2024 | Non-original (review/editorial/abstract) | Non‑original article (review/editorial/abstract); not eligible. |
| Rosen [52] | 2021 | No extractable 2×2 or HR (ROC/AUC or qualitative only) | Reported ROC/AUC or qualitative results only; no per‑patient 2×2 or HR with 95% CI available. |
| Schwarzenberg [124] | 2012 | Not eligible per protocol | Not eligible per prespecified protocol criteria. |
| Shishido [125] | 2012 | No extractable 2×2 or HR (ROC/AUC or qualitative only) | Reported ROC/AUC or qualitative results only; no per‑patient 2×2 or HR with 95% CI available. |
| Song [126] | 2020 | No extractable 2×2 or HR (ROC/AUC or qualitative only) | Reported ROC/AUC or qualitative results only; no per‑patient 2×2 or HR with 95% CI available. |
| Tripathi [127] | 2012 | No extractable 2×2 or HR (ROC/AUC or qualitative only) | Reported ROC/AUC or qualitative results only; no per‑patient 2×2 or HR with 95% CI available. |
| Verger [128] | 2018 | Not HGG-only or mixed-grade not separable | Population mixed or not high‑grade‑only; HGG subset not separable for extraction. |
| Wardak [129] | 2014 | Interventional PET-guided study (not eligible) | Interventional PET‑guided design (monitoring or PET‑guided resection/therapy); not eligible per protocol. |
| Xiong [130] | 2024 | No extractable 2×2 or HR (ROC/AUC or qualitative only) | Reported ROC/AUC or qualitative results only; no per‑patient 2×2 or HR with 95% CI available. |
| Yoo [131] | 2015 | Not HGG-only or mixed-grade not separable | Population mixed or not high‑grade‑only; HGG subset not separable for extraction. |
| Zaragori [132] | 2020 | No extractable 2×2 or HR (ROC/AUC or qualitative only) | Reported ROC/AUC or qualitative results only; no per‑patient 2×2 or HR with 95% CI available. |
| Zaragori [44] | 2021 | No extractable 2×2 or HR (ROC/AUC or qualitative only) | Reported ROC/AUC or qualitative results only; no per‑patient 2×2 or HR with 95% CI available. |
| Zaragori [43] | 2022 | No extractable 2×2 or HR (ROC/AUC or qualitative only) | Reported ROC/AUC or qualitative results only; no per‑patient 2×2 or HR with 95% CI available. |
| Zhou [45] | 2024 | No extractable 2×2 or HR (ROC/AUC or qualitative only) | Reported ROC/AUC or qualitative results only; no per‑patient 2×2 or HR with 95% CI available. |

**Supplementary Table S2. Full‑text articles excluded at eligibility assessment**
