# Supplementary material for: Metabolic Heterogeneity in High-Grade Glioma Assessed by Multi-Tracer PET and Ex Vivo Metabolomics: A Systematic Review and Meta-Analysis
Source: Metabolites. 2025 Dec 24;16(1):17. doi: 10.3390/metabo16010017 (PMC12844024; doi:10.3390/metabo16010017)
Supplement: Supplementary file 1 [file metabolites-16-00017-s001.zip › Table S3. QUADAS-2 (diagnostic accuracy)..docx]

**Table S3**. QUADAS-2 (diagnostic accuracy).

Domain-level risk-of-bias and applicability judgments for diagnostic studies (PsP and TRC). Domains: Patient selection; Index test; Reference standard; Flow/timing; plus applicability. Symbols: 🟢 Low; 🟡 Unclear; 🔴 High.

| **Study (tracer)** | **Patient selection (RoB)** | **Index test (RoB)** | **Reference standard (RoB)** | **Flow & timing (RoB)** | **Patient selection (Applic.)** | **Index test (Applic.)** | **Ref. standard (Applic.)** |
| --- | --- | --- | --- | --- | --- | --- | --- |
| Pellerin 2021 (FDOPA) [23] | 🟢 | 🟡 | 🟢 | 🟡 | 🔴 | 🟢 | 🟢 |
| Nabavizadeh 2023 (Fluciclovine) [24] | 🟢 | 🔴 | 🟢 | 🟢 | 🟢 | 🟢 | 🟢 |
| Herrmann 2014 (FDOPA) [25] | 🔴 | 🔴 | 🟡 | 🟡 | 🟢 | 🟢 | 🟢 |
| Karunanithi 2013 (FDOPA) [26] | 🔴 | 🟡 | 🟡 | 🟡 | 🟢 | 🟢 | 🟢 |
| Khangembam 2014 (FDG) [27] | 🔴 | 🟡 | 🟡 | 🟡 | 🟢 | 🟢 | 🟢 |
| Khangembam 2014 (¹³N-NH₃) [27] | 🔴 | 🟡 | 🟡 | 🟡 | 🟢 | 🟢 | 🟢 |
