# Supplementary material for: Metabolic Heterogeneity in High-Grade Glioma Assessed by Multi-Tracer PET and Ex Vivo Metabolomics: A Systematic Review and Meta-Analysis
Source: Metabolites. 2025 Dec 24;16(1):17. doi: 10.3390/metabo16010017 (PMC12844024; doi:10.3390/metabo16010017)
Supplement: Supplementary file 1 [file metabolites-16-00017-s001.zip › Table S4. QUIPS (prognosis OS and PFS)..docx]

**Table S4**. QUIPS (prognosis: OS and PFS).

Domain-level QUIPS judgments for prognostic cohorts (OS and PFS). Domains: Study participation; Study attrition; Prognostic-factor measurement; Outcome measurement; Confounding; Analysis & reporting. Symbols: 🟢 Low; 🟡 Moderate/Some concerns; 🔴 High; ⚪ Unclear.

| **Study** | **Participation** | **Attrition** | **Prognostic factor measurement** | **Outcome measurement** | **Confounding** | **Analysis & reporting** |
| --- | --- | --- | --- | --- | --- | --- |
| Colavolpe 2012 (FDG, HGG) [28] | 🟡 | 🟢 | 🟢 | 🟢 | 🟢 | 🟢 |
| Leiva-Salinas 2017 (FDG PET/MR, GBM prog.) [29] | 🟡 | 🟢 | 🟢 | 🟢 | ⚪ | 🟡 |
| Chiang 2017 (FDG, metabolic tumor size) [30] | 🟡 | 🟢 | 🟡 | 🟢 | ⚪ | 🟡 |
| Graham 2020 (FDG, BEV) [31] | 🟡 | 🟢 | 🟢 | 🟢 | 🟢 | 🔴 |
| Jansen 2015 (FET dynamic, HGG) [32] | 🟡 | 🟢 | 🟢 | 🟢 | 🟡 | 🟡 |
| Suchorska 2015 (FET, GBM, prospective) [33] | 🟢 | 🟢 | 🟢 | 🟢 | 🟢 | 🟢 |
| Bauer 2020 (FET dynamic, IDH-wt) [34] | 🟡 | 🟢 | 🟢 | 🟢 | 🟢 | 🟢 |
| Wirsching 2021 (FET, ARTE RCT) [35] | 🟢 | 🟢 | 🟢 | 🟢 | 🟢 | 🟢 |
| Zhao 2014 (FLT, recurrent) [36] | 🟡 | 🟢 | 🟢 | 🟢 | 🔴 | 🟡 |
| Gerstner 2016 (FMISO, ACRIN 6684) [11] | 🟢 | 🟡 | 🟢 | 🟢 | 🔴 | 🟡 |
| Miller 2020 (¹¹C-MET) [38] | 🟡 | 🟡 | 🟢 | 🟢 | 🟡 | 🟡 |
| Huang 2021 (¹⁸F-FMISO) [39] | 🟡 | 🟢 | 🟢 | 🟢 | 🔴 | 🔴 |
| Rozenblum 2023 (FDOPA) [37] | 🟡 | 🟢 | 🟢 | 🟢 | 🟡 | 🟡 |

**Common concerns: patient selection and post-hoc thresholds; reference standard and flow/timing generally acceptable. Confounding varied in prognostic cohorts.**
