# Supplementary material for: Metabolic Heterogeneity in High-Grade Glioma Assessed by Multi-Tracer PET and Ex Vivo Metabolomics: A Systematic Review and Meta-Analysis
Source: Metabolites. 2025 Dec 24;16(1):17. doi: 10.3390/metabo16010017 (PMC12844024; doi:10.3390/metabo16010017)
Supplement: Supplementary file 1 [file metabolites-16-00017-s001.zip › Table S5. Per-patient 2脳2 .docx]

| **Study_ID** | **Tracer** | **Outcome** | **N_total** | **TP** | **FP** | **FN** | **TN** | **Any_zero_cell** | **Note_on_reference_standard** |
| --- | --- | --- | --- | --- | --- | --- | --- | --- | --- |
| Pellerin_2021 [23] | ¹⁸F-FDOPA | PsP vs progression (per-patient) | 48 | 29 | 3 | 2 | 14 | No | Histology and/or RANO/Macdonald follow-up |
| Nabavizadeh_2023 [24] | ¹⁸F-FACBC | PsP vs progression (per-patient) | 28 | 21 | 1 | 1 | 5 | No | Histology and/or RANO/Macdonald follow-up |
| Herrmann_2014 [25] | ¹⁸F-FDOPA | Recurrence/progression vs TRC | 110 | 69 | 8 | 12 | 21 | No | Histology and/or RANO/Macdonald follow-up |
| Karunanithi_2013 [26] | ¹⁸F-FDOPA | Recurrence/progression vs TRC | 24 | 18 | 0 | 0 | 6 | Yes | Histology and/or RANO/Macdonald follow-up |
| Khangembam_2014_FDG [27] | ¹⁸F-FDG | Recurrence/progression vs TRC | 18 | 7 | 3 | 2 | 6 | No | Histology and/or RANO/Macdonald follow-up |
| Khangembam_2014_NH3 [27] | ¹³N-NH₃ | Recurrence/progression vs TRC | 18 | 7 | 3 | 2 | 6 | No | Histology and/or RANO/Macdonald follow-up |

Table S5. Per-patient 2×2 diagnostic datasets for PsP and recurrence/progression versus treatment-related change (TRC) in HGG (WHO III–IV). Raw counts are reported as extracted; presence of zero cells is flagged. Aggregated totals and continuity corrections (when applied for variance estimation) are described in the Methods.
