# Supplementary material for: Metabolic Heterogeneity in High-Grade Glioma Assessed by Multi-Tracer PET and Ex Vivo Metabolomics: A Systematic Review and Meta-Analysis
Source: Metabolites. 2025 Dec 24;16(1):17. doi: 10.3390/metabo16010017 (PMC12844024; doi:10.3390/metabo16010017)
Supplement: Supplementary file 1 [file metabolites-16-00017-s001.zip › Table S6. Extracted hazard ratios (HRs).docx]

Table S6. Extracted hazard ratios (HRs), confidence intervals and adjustment covariates for OS and PFS in HGG (WHO grade III–IV). For each study we list the PET metric retained for meta-analysis according to a prespecified hierarchy, the type of model (univariable versus multivariable) and the main covariates. Only one HR per study–endpoint was retained for quantitative pooling.

| **Study_ID** | **Tracer** | **Outcome** | **PET_metric** | **HR** | **CI_lower** | **CI_upper** | **Model_type** | **Covariates** | **N_total_HGG** | **Included_in_meta** |
| --- | --- | --- | --- | --- | --- | --- | --- | --- | --- | --- |
| Bauer 2020 [34] | ¹⁸F-FET | OS | TTP (min) | 0.941 | 0.90 | 0.984 | Multivariable (Cox) | MGMT methylated; KPS = 100%; complete resection; (TBRmax, TBRmean, slope, MTV included) | 45 | Yes |
| Jansen 2015 [32] | ¹⁸F-FET | OS | TTPmin (≤12.5 vs >12.5 min) | 2.04 | 1.2 | 3.48 | Multivariable (Cox) | Alternative models with WHO grade or TTPmin (see text) | 111* | Yes |
| Colavolpe 2012 [28] | ¹⁸F-FDG | OS | TBRmax (lesion / contralateral cortex ratio, continuous) | 2.36 | 1.01 | 5.53 | Multivariable (Cox) | Age; sex; KPS; location; initial treatment; number of previous treatment lines; corticosteroid use | 25 | Yes |
| Rozenblum 2023 [37] | ¹⁸F-FDOPA | PFS | TBRmean (continuous) | 7.92 | 2.17 | 28.9 | Multivariable (Cox) | Multivariable analysis restricted to significant covariates (≥2 FDOPA-avid needles; TBRmean; MTV; WHO grade; other factors considered in the full global PFS model: WHO grade, TBRmean, MTV, slope) | 90 | Yes |
| Gerstner 2016 [11] | ¹⁸F-FMISO | OS | SUVmax (hypoxic volume) | 1.16 | 0.75 | 1.81 | Multivariable (Cox) | — | 38 | Yes |
| Huang 2021 [39] | ¹⁸F-FMISO | PFS | Hypoxic volume (HV, continuous) | 1.67 | 1.14 | 2.45 | Multivariable (Cox) | — | 33 | Yes |
| Graham 2020 [31] | ¹⁸F-FDG | OS | SUVmax (continuous) | 1.07 | 0.95 | 1.20 | Multivariable (Cox) | Age; sex; KPS; MGMT status (model also including FDG avidity) | 31 | Yes |
| Suchorska 2015 [33] | ¹⁸F-FET | OS | BTV (mL) | 1.028 | 1.008 | 1.049 | Multivariable (Cox) | Age; performance status; multiple vs single lesions; lesion size (MRI volume); initial TAC pattern (decreasing vs increasing) | 79 | Yes |
| Leiva-Salinas 2017 [29] | ¹⁸F-FDG | OS | Metabolic tumour size (cm, continuous) | 1.11 | 1.03 | 1.20 | Multivariable (Cox) | Age; sex; KPS; type of surgery (GTR vs biopsy); tumour size; location | 56 | Yes |
| Chiang 2017 [30] | ¹⁸F-FDG | OS | Metabolic tumour size (cm, continuous) | 1.07 | 1.01 | 1.14 | Multivariable (Cox) | Age; sex; KPS; adjuvant treatment; type of initial resection (GTR vs partial resection) | 44 | Yes |
| Wirsching 2021 [35] | ¹⁸F-FET | OS | “High-risk” FET uptake pattern (categorical) | 1.71 | 0.70 | 4.20 | Multivariable (Cox) | Age; KPS; treatment; standard clinical variables; interaction corrections for treatment arm | 31 | Yes |
| Zhao 2014 [36] | ¹⁸F-FLT | OS | SUVmax (continuous) | 1.18 | 1.13 | 1.24 | Multivariable (Cox) | Significant univariable predictors included: KPS, grade, tumour size, PGV (in the final model PGV and T/N remained independent) | 56 | Yes |
| Miller 2020 [38] | ¹¹C-MET | PFS | Baseline MTV (mL, continuous) | 1.6 | 1.2 | 2.3 | Univariable (Cox) | — | 37 | Yes |
