# Supplementary material for: Metabolic Heterogeneity in High-Grade Glioma Assessed by Multi-Tracer PET and Ex Vivo Metabolomics: A Systematic Review and Meta-Analysis
Source: Metabolites. 2025 Dec 24;16(1):17. doi: 10.3390/metabo16010017 (PMC12844024; doi:10.3390/metabo16010017)
Supplement: Supplementary file 1 [file metabolites-16-00017-s001.zip › Table S8. PRISMA Checklist.docx]

# PRISMA Abstract Checklist

| **Topic** | **No.** | **Item** | **Location where item is reported** |
| --- | --- | --- | --- |
| **TITLE** |  |  |  |
| **Title** | 1 | Identify the report as a systematic review. | Title page: “Metabolic heterogeneity in high-grade glioma … a systematic review and meta-analysis.” |
| **ABSTRACT** |  |  |  |
| **Abstract** | 2 | See the PRISMA 2020 for Abstracts checklist |  |
| **INTRODUCTION** |  |  |  |
| **Rationale** | 3 | Describe the rationale for the review in the context of existing knowledge. | Introduction, first paragraphs (clinical gap: MRI limits; PET multi-tracer rationale; unmet need for imaging-to-omics). |
| **Objectives** | 4 | Provide an explicit statement of the objective(s) or question(s) the review addresses. | End of Introduction: aims cover diagnostic (PsP/TRC), prognostic (OS/PFS), molecular markers, and PET–ex-vivo concordance; PROSPERO ID stated. |
| **METHODS** |  |  |  |
| **Eligibility criteria** | 5 | Specify the inclusion and exclusion criteria for the review and how studies were grouped for the syntheses. | Methods → “Eligibility.” Adults with HGG (WHO III–IV). Diagnostic pooling: per-patient 2×2 for PsP and TRC (exclude per-lesion/mixed non-separable). Prognostic pooling: HR with 95% CI from baseline PET; exclude PET-guided interventional studies if baseline effect inseparable; general exclusions listed (LGG-only, pediatric-only, preclinical, reviews/case reports, missing key stats). |
| **Information sources** | 6 | Specify all databases, registers, websites, organisations, reference lists and other sources searched or consulted to identify studies. Specify the date when each source was last searched or consulted. | Methods → “Information sources and search.” Databases: PubMed/MEDLINE and Web of Science Core Collection (Clarivate); inception to 2025-09-01; backward/forward citation chasing; trial registries (ClinicalTrials.gov, WHO ICTRP). |
| **Search strategy** | 7 | Present the full search strategies for all databases, registers and websites, including any filters and limits used. | Methods → “Information sources and search”; Supplementary Table S1 provides the full reproducible queries for PubMed and WoS (fields/operators/limits)”. |
| **Selection process** | 8 | Specify the methods used to decide whether a study met the inclusion criteria of the review, including how many reviewers screened each record and each report retrieved, whether they worked independently, and if applicable, details of automation tools used in the process. | Methods → “Study selection followed PRISMA 2020”; PRISMA flow diagram Figure 1; Supplementary Table S2 lists full-text exclusions with primary reason (deduplicated). |
| **Data collection process** | 9 | Specify the methods used to collect data from reports, including how many reviewers collected data from each report, whether they worked independently, any processes for obtaining or confirming data from study investigators, and if applicable, details of automation tools used in the process. | Methods → “Data extraction.” Two reviewers; piloted template; identifiers, PET metrics/cut-offs, reference standard; diagnostic TP/FP/FN/TN; prognostic HR(95%CI)/adjustment set; molecular AUC; ex-vivo variables. Preference rules (per-patient over per-lesion; one effect per study–endpoint). No imputation. |
| **Data items** | 10a | List and define all outcomes for which data were sought. Specify whether all results that were compatible with each outcome domain in each study were sought (e.g. for all measures, time points, analyses), and if not, the methods used to decide which results to collect. | Methods → endpoints: diagnostic PsP and TRC; prognostic OS/PFS; molecular markers (IDH, MGMT, 1p/19q, ATRX, TERT); ex-vivo concordance. |
|  | 10b | List and define all other variables for which data were sought (e.g. participant and intervention characteristics, funding sources). Describe any assumptions made about any missing or unclear information. | Methods → definitions of PET metrics (SUV, TBR, MTV, TLG, dynamic TTP), tracer classes; prespecified metric hierarchy per tracer; adjustment covariates (age, PS, MGMT, ±EOR). |
| **Study risk of bias assessment** | 11 | Specify the methods used to assess risk of bias in the included studies, including details of the tool(s) used, how many reviewers assessed each study and whether they worked independently, and if applicable, details of automation tools used in the process. | Methods → “Risk of bias.” Tools: QUADAS-2 (diagnostic) and QUIPS (prognostic), two reviewers, consensus; details in Supplementary Tables S3–S4 (traffic-light). Sensitivity analyses informed by these judgments. |
| **Effect measures** | 12 | Specify for each outcome the effect measure(s) (e.g. risk ratio, mean difference) used in the synthesis or presentation of results. | Methods → “Effect measures and synthesis.” Diagnostic: sensitivity/specificity (HSROC where feasible). Prognostic: log-HR with random-effects (REML, Hartung–Knapp). Molecular: AUC. |
| **Synthesis methods** | 13a | Describe the processes used to decide which studies were eligible for each synthesis (e.g. tabulating the study intervention characteristics and comparing against the planned groups for each synthesis (item 5)). | Methods → choice rules (one effect per study–endpoint; per-patient preferred; non-overlapping cohorts; biologically coherent strata). |
|  | 13b | Describe any methods required to prepare the data for presentation or synthesis, such as handling of missing summary statistics, or data conversions. | Methods → no imputation; harmonised definitions; deduplication of records before screening; metric hierarchy per tracer. |
|  | 13c | Describe any methods used to tabulate or visually display results of individual studies and syntheses. | Methods → random-effects with HK adjustment; HSROC/bivariate planned when k≥3; otherwise descriptive. Stratification by tracer class; avoid incoherent pooling for PFS. |
|  | 13d | Describe any methods used to synthesize results and provide a rationale for the choice(s). If meta-analysis was performed, describe the model(s), method(s) to identify the presence and extent of statistical heterogeneity, and software package(s) used. | Methods/Results → heterogeneity acknowledged; I² reported in OS summary; sensitivity analyses (exclude high-risk QUADAS-2/QUIPS; metric-based). |
|  | 13e | Describe any methods used to explore possible causes of heterogeneity among study results (e.g. subgroup analysis, meta-regression). | Methods → prespecified; Results → examples (exclude dynamic FET TTP; RoB-informed). |
|  | 13f | Describe any sensitivity analyses conducted to assess robustness of the synthesized results. | Methods → planned exploratory meta-regression by tracer where k≥3; not performed where strata too small. |
| **Reporting bias assessment** | 14 | Describe any methods used to assess risk of bias due to missing results in a synthesis (arising from reporting biases). | Methods → small-study tests not performed when k<10; narrative consideration of selective reporting; explicitly stated in synthesis notes. |
| **Certainty assessment** | 15 | Describe any methods used to assess certainty (or confidence) in the body of evidence for an outcome. | Not performed (not applicable); justification: diverse designs, small k in strata, heterogeneous metrics; addressed instead via QUADAS-2/QUIPS and sensitivity analyses (state “Not assessed; see Risk of bias & Sensitivity analyses”). |
| **RESULTS** |  |  |  |
| **Study selection** | 16a | Describe the results of the search and selection process, from the number of records identified in the search to the number of studies included in the review, ideally using a flow diagram. | Results + Figure 1 (PRISMA flow); counts per step reported; diagnostic/prognostic arms described. Supplementary Table S2 lists full-text exclusions with reasons. |
|  | 16b | Cite studies that might appear to meet the inclusion criteria, but which were excluded, and explain why they were excluded. | Supplementary Table S2 — “Full-text articles excluded with primary reason” (deduplicated; with PMIDs where available). |
| **Study characteristics** | 17 | Cite each included study and present its characteristics. | Results → Diagnostic Tables 1–2 (per-patient 2×2, tracers/endpoints); Prognostic Table 3 (OS/PFS); Molecular Tables 4–5; Ex-vivo Table 6. |
| **Risk of bias in studies** | 18 | Present assessments of risk of bias for each included study. | Results → brief summary in text; full domain-level judgments in Supplementary S3 (QUADAS-2) and S4 (QUIPS). |
| **Results of individual studies** | 19 | For all outcomes, present, for each study: (a) summary statistics for each group (where appropriate) and (b) an effect estimate and its precision (e.g. confidence/credible interval), ideally using structured tables or plots. | Diagnostic: Tables 1–2 (TP/FP/FN/TN; Se/Sp). Prognostic: Table 3 and Figure 2 (HRs, 95%CIs). Molecular: Table 4 (per-study performance). |
| **Results of syntheses** | 20a | For each synthesis, briefly summarise the characteristics and risk of bias among contributing studies. | Results → PsP pooled Se/Sp; TRC FDOPA pooled Se/Sp; OS pooled HRs for FDG and AA (with HK); PFS study-level HRs; uncertainty and heterogeneity reported. |
|  | 20b | Present results of all statistical syntheses conducted. If meta-analysis was done, present for each the summary estimate and its precision (e.g. confidence/credible interval) and measures of statistical heterogeneity. If comparing groups, describe the direction of the effect. | Results → I² and prediction intervals for OS; narrative heterogeneity for PFS; tracer/metric stratification explained. |
|  | 20c | Present results of all investigations of possible causes of heterogeneity among study results. | Results → HK vs DL; excluding dynamic metrics; RoB-informed exclusions—no material change to conclusions. |
|  | 20d | Present results of all sensitivity analyses conducted to assess the robustness of the synthesized results. | Not conducted (insufficient k within strata); stated under Methods; Results note rationale. |
| **Reporting biases** | 21 | Present assessments of risk of bias due to missing results (arising from reporting biases) for each synthesis assessed. | Results → Not assessed via formal tests (k<10); addressed narratively; see Limitations. |
| **Certainty of evidence** | 22 | Present assessments of certainty (or confidence) in the body of evidence for each outcome assessed. | Not graded (no GRADE); overall confidence discussed qualitatively in Discussion (heterogeneity, retrospective base). |
| **DISCUSSION** |  |  |  |
| **Discussion** | 23a | Provide a general interpretation of the results in the context of other evidence. | Discussion → “Key findings” and integration with prior work (diagnostic superiority of AA-PET for PsP/TRC; weak/heterogeneous baseline prognostic value). |
|  | 23b | Discuss any limitations of the evidence included in the review. | Discussion → heterogeneity (metrics, acquisition), small k, retrospective predominance; risk of selection and threshold bias. |
|  | 23c | Discuss any limitations of the review processes used. | Discussion/Limitations → inability to run HSROC in small strata; no formal publication bias tests (k<10); narrative ex-vivo concordance due to lack of co-registered maps. |
|  | 23d | Discuss implications of the results for practice, policy, and future research. | Discussion → clinical implications (use AA-PET post-CRT; report SUV/TBR and MTV/BTV) and research roadmap (EARL harmonisation, RANO 2.0 alignment, prospective PET/MR + stereotactic ex-vivo pipelines). |
| **OTHER INFORMATION** |  |  |  |
| **Registration and protocol** | 24a | Provide registration information for the review, including register name and registration number, or state that the review was not registered. | Methods — Registration: PROSPERO (International Prospective Register of Systematic Reviews), CRD420251113416. |
|  | 24b | Indicate where the review protocol can be accessed, or state that a protocol was not prepared. | Declarations — Protocol: No standalone protocol was prepared beyond the PROSPERO record. |
|  | 24c | Describe and explain any amendments to information provided at registration or in the protocol. | Declarations — Protocol amendments: None; no amendments were made to the registered information or protocol after registration |
| **Support** | 25 | Describe sources of financial or non-financial support for the review, and the role of the funders or sponsors in the review. | Back matter → Funding: “This research received no external funding |
| **Competing interests** | 26 | Declare any competing interests of review authors. | Back matter → “The authors declare no conflicts of interest. |
| **Availability of data, code and other materials** | 27 | Report which of the following are publicly available and where they can be found: template data collection forms; data extracted from included studies; data used for all analyses; analytic code; any other materials used in the review. | Back matter → Data Availability Statement (current): “This meta-analysis used only study-level data extracted from published articles; no individual participant data were collected. The curated extraction spreadsheets (2×2 tables and hazard ratios), PRISMA search strategies (Table S1) and selection logs (Table S2) are available from the corresponding author upon reasonable request. PROSPERO: CRD420251113416.…” |

#####

# PRIMSA

| **Topic** | **No.** | **Item** | **Reported?** |
| --- | --- | --- | --- |
| **TITLE** |  |  |  |
| **Title** | 1 | Identify the report as a systematic review. | Yes |
| **BACKGROUND** |  |  |  |
| **Objectives** | 2 | Provide an explicit statement of the main objective(s) or question(s) the review addresses. | Yes |
| **METHODS** |  |  |  |
| **Eligibility criteria** | 3 | Specify the inclusion and exclusion criteria for the review. | Yes |
| **Information sources** | 4 | Specify the information sources (e.g. databases, registers) used to identify studies and the date when each was last searched. | Yes |
| **Risk of bias** | 5 | Specify the methods used to assess risk of bias in the included studies. | Yes |
| **Synthesis of results** | 6 | Specify the methods used to present and synthesize results. | Yes |
| **RESULTS** |  |  |  |
| **Included studies** | 7 | Give the total number of included studies and participants and summarise relevant characteristics of studies. | Yes |
| **Synthesis of results** | 8 | Present results for main outcomes, preferably indicating the number of included studies and participants for each. If meta-analysis was done, report the summary estimate and confidence/credible interval. If comparing groups, indicate the direction of the effect (i.e. which group is favoured). | Yes |
| **DISCUSSION** |  |  |  |
| **Limitations of evidence** | 9 | Provide a brief summary of the limitations of the evidence included in the review (e.g. study risk of bias, inconsistency and imprecision). | Yes |
| **Interpretation** | 10 | Provide a general interpretation of the results and important implications. | Yes |
| **OTHER** |  |  |  |
| **Funding** | 11 | Specify the primary source of funding for the review. | Yes |
| **Registration** | 12 | Provide the register name and registration number. | Yes |

*From:* Page MJ, McKenzie JE, Bossuyt PM, Boutron I, Hoffmann TC, Mulrow CD, et al. The PRISMA 2020 statement: an updated guideline for reporting systematic reviews. MetaArXiv. 2020, September 14. DOI: 10.31222/osf.io/v7gm2. For more information, visit: [www.prisma-statement.org](file:///Users/zulien_tod/Downloads/www.prisma-statement.org)
